# Physical functions assessed by lower limb performance-based and self-reported outcome measures for knee musculoskeletal conditions: A scoping review

**DOI:** 10.1016/j.bjpt.2024.101166

**Published:** 2024-12-12

**Authors:** Andrés Pierobon, Will Taylor, Richelle Caya, Federico Villalba, Santiago Soliño, Pablo Oscar Policastro, Richard Siegert, Ben Darlow

**Affiliations:** a University of Otago, Wellington, New Zealand; b University of Otago, Dunedin, New Zealand; c Santojanni Hospital, Buenos Aires, Argentina; d Durand Hospital, Buenos Aires, Argentina; e University of San Carlos, San Carlos, Brazil; f Auckland University of Technology, Auckland, New Zealand

**Keywords:** Anterior cruciate ligament injuries, Knee osteoarthritis, Patellofemoral pain, Patient reported outcome measures, Physical functional performance, Psychometrics

## Abstract

**Background:**

Physical function assessment is key for the management of knee musculoskeletal conditions. There are a wide variety of self-reported outcome measures (SROMs) and performance-based outcome measures (PBOMs) to assess physical function of individuals with knee conditions. However, the content of these measures has not been explored.

**Objective:**

To explore the range and frequency of physical functions assessed by lower limb PBOMs and SROMs for people with knee osteoarthritis (OA), anterior cruciate ligament (ACL) injuries, and patellofemoral pain (PFP).

**Methods:**

A scoping review was conducted. We included development or measurement properties studies of knee functional outcome measures for populations with knee OA, ACL injuries, and PFP. We extracted the physical functions assessed in each measure. Each identified physical function was linked to a code from the International Classification of Functioning, Disability and Health (ICF) framework.

**Results:**

4146 articles were screened. A total of 143 articles were included. The median number of physical functions assessed was nine for SROMs and one for PBOMs. The three most assessed physical functions were climbing stairs, walking short distances, and standing up from sitting. Climbing stairs was the most assessed physical function in measures for knee OA and PFP populations, whereas jumping was in measures for the ACL-injured population.

**Conclusion:**

SROMs assess a broader range of physical functions, whereas PBOMs focus on discrete activities. ACL and PFP measures evaluated more challenging physical functions than knee OA measures. Current physical function outcome measures are not well suited to assess performance in knee OA populations with mild or diverse levels of impairment.

## Highlights

• Climbing stairs was the most assessed physical function.
• Lower limb physical functions performance tests assess a limited number of activities.
• Knee OA outcome measures rarely assess challenging activities like running or jumping.
• Some issues related to the drafting of self-reported items were found.

## Introduction

Knee musculoskeletal (MSK) conditions significantly affect activities of daily living and physical performance in both the athletic and non-athletic populations.^1^, ^2^, ^3^ The assessment of physical function is key for the management of these conditions.^4^^,^^5^ Effective physical function measurement is important for assessing the burden of disease in a specific population, assessing the efficacy of different interventions in research, and tracking clinical intervention effectiveness and patient progress.^6^ Several measures of lower limb physical function have been developed and are available for people with knee MSK conditions, but many have poor psychometric properties, limited applicability for key populations (e.g., early knee osteoarthritis [OA]), and a narrow scope of assessment (i.e., performance tests that assess a single activity).^5^^,^^7^, ^8^, ^9^

Physical function, defined by the International Classification of Functioning, Disability and Health (ICF) as “the ability to move around and perform daily activities”, is complex to evaluate. Physical function represents multiple constructs such as different components (e.g. joint mobility, strength, balance, power), activity capacity (e.g. walking, climbing steps, kneeling, squatting) and ability to participate (e.g. employment, sports, hiking, gardening, playing with grandchildren).^10^ There is no gold standard for the assessment of lower limb physical function.^5^ There is a wide variety of self-reported outcome measures (SROMs) and performance-based outcome measures (PBOMs).^11^, ^12^, ^13^ SROMs and PBOMs measure different aspects of physical function, each with advantages and disadvantages.^14^^,^^15^ SROMs assess respondents’ perceptions of ability or limitation, whereas PBOMs objectively assess physical performance.^16^ SROMs tend to be less time-consuming to administer, can usually be completed independently by respondents, and are useful for conditions with fluctuating symptoms allowing the assessment of longer periods of time and average performance. However, SROM respondents may overestimate or underestimate their functional capacity, reducing the accuracy of the information collected.^17^ Self-reported data can also be affected by expectation, social desirability, and recall bias.^18^ These biases reduce the strength of evidence generated about interventions assessed by SROMs.^19^ In contrast, PBOMs have fewer biases (no recall bias or estimation bias, but may have measurement errors) and are generally more reproducible and reliable evaluations of activities of daily living and physical performance.^20^ In addition, PBOMs have greater sensitivity to change and less vulnerability to external influences such as cognition, culture, language, and education.^21^ The limitations of PBOMs include the assessment of a limited number of physical functions, the necessity of a competent observer for assessing and scoring, and higher time requirements than SROMs.^22^, ^23^, ^24^

Published systematic reviews of knee functional measures have studied the quality of the development process and the psychometric properties in specific populations (e.g., performance tests in people with anterior cruciate ligament [ACL] injuries).^25^, ^26^, ^27^ However, to our knowledge, no study has explored the *content* of these measures (i.e., which or how many physical functions are included). Identifying physical functions common to different measures will allow a better understanding of the spectrum of physical functions assessed and will help to identify gaps to address when developing new measures.

We aimed to explore the spectrum of physical functions assessed and how often they are assessed in existing SROMs and PBOMs used to measure knee function and physical performance in people with knee MSK conditions. Our secondary objectives were to compare the range and frequency of physical functions assessed in 1) different populations, and 2) different types of measure (PBOMs and SROMs).

## Methods

We conducted a scoping review of the literature. Scoping reviews map key concepts underpinning a particular area of research and explore broad areas to identify gaps in the evidence, clarify concepts, and inform of the type of evidence particular to a research topic. A systematic review is well suited to address more precisely focused questions like assessing the psychometric properties of a specific measurement instrument but less suited to summarising a heterogeneous body of knowledge or identifying gaps in existing literature.^28^ This scoping review followed The Joanna Briggs Institute (JBI) Reviewers’ Manual 2015 and is reported according to Preferred Reporting Items for Systematic Reviews and Meta-Analyses–Scoping Review (PRISMA-ScR) guidelines.^28^^,^^29^ The protocol is published in the Open Science Framework.^30^

### Stage one: identifying the research question

The research question was defined as: *‘What are the physical functions assessed by existing PBOMs and SROMs used to measure knee function and physical performance in people with knee OA, ACL injury, or patellofemoral pain (PFP)?’* These populations were selected as they are three of the most common knee MSK conditions and can be considered representative of most knee problems.^2^^,^^4^^,^^31^

### Stage two: identifying relevant studies

#### Inclusion criteria

Study designs: We included peer-reviewed studies describing the development or the assessment of measurement properties of outcome measures. We use the COSMIN (COnsensus-based Standards for the selection of health status Measurement INstruments) taxonomy to define a measurement properties’ study.^32^

Target population: We included studies involving participants with knee OA, ACL injury, or PFP.

Construct: We included studies of PBOMs or SROMs that measure lower limb ´physical function´ (defined as the ability to move around and perform daily activities) or athletic performance.^33^ PBOMs were defined as “*a measurement based on a task(s) performed by a patient, according to instructions, that is administered and evaluated by a health care professional/researcher”.**^16^* SROMs were defined as "*a measurement based on a report that comes directly from the patient about the status of a patient's health condition without amendment or interpretation of the patient's response by a clinician or anyone else”.**^16^*

Language: We included studies published in English.

#### Exclusion criteria

We excluded studies published in the ´grey literature´ (e.g., scientific meeting abstracts, dissertations, or theses), those not electronically available, translations and cross-cultural adaptation studies or studies using translated or cross-culturally adapted versions of existing measures, studies including qualitative performance tests (e.g., tests assessing knee valgus, gait analysis), and measures assessing generic physical function without a specific lower limb subscale.

#### Data sources and search strategy

Relevant studies were identified by searching electronic databases (Medline, CINAHL, Scopus, and Web of Science) from inception. The search was conducted on February 7th, 2023. The search strategy was developed iteratively with the help of a reference librarian and followed three key steps:

Step 1: Initial limited search. We conducted an initial search in Medline and CINAHL databases.
Step 2: Identify keywords and index terms. We analysed the text words contained in the title and abstract of the papers retrieved and used these terms and keywords for a second search in Web of Science and Scopus databases.
Step 3: Execution of final search strategy and further searching of references and citations of the included articles.

#### Key terms

The search strategy combined four components (target population, outcome measure, construct, measurement properties) and some exclusion terms. Table 1 describes the search strategy for the Medline database. The complete search strategy can be found in the Supplementary material 1.

**Table 1:** Search strategy for Medline database.

SET #	Keywords and operators
1	(knee osteoarthritis.mp. or Osteoarthritis, Knee/ OR knee OA.mp. OR osteoarthritis of the knee.mp. OR knee arthritis.mp. OR arthritis of the knee.mp. OR Anterior Cruciate Ligament Injuries/ or Anterior Cruciate Ligament/ or anterior cruciate ligament.mp. OR Anterior Cruciate Ligament Reconstruction/ or ACL.mp. OR Patellofemoral Pain Syndrome/ or patellofemoral knee pain syndrome.mp. OR patellofemoral pain syndrome.mp. OR patellofemoral pain.mp. OR PFPS.mp.) NOT (knee replacement.m_titl. OR joint replacement.m_titl. OR "arthroplast*".m_titl. OR meniscectomy.m_titl. OR "surg*".m_titl. OR "arthroscop*".m_titl. OR "corticoid*".m_titl.).
2	outcome measure.mp. or Outcome Assessment, Health Care/ OR questionnaire.mp. or "Surveys and Questionnaires"/ OR test*.mp. OR performance based*.mp. OR performance measure.mp. OR performance instrument.mp. OR performance scale.mp. OR performance index.mp. OR Patient Reported Outcome Measures/ or patient reported*.mp. OR self report*.mp. OR observation measure.mp.
3	(performance.mp. or Athletic Performance/ OR Disability Evaluation/ or "International Classification of Functioning, Disability and Health"/ or disability.mp. OR "Activities of Daily Living"/ or physical function.mp. OR functionality.mp. OR function.mp. OR return to sport.mp. or Return to Sport/ OR physical activity.mp. or Exercise/ OR functional activity.mp. OR deficit.mp. OR "Activities of Daily Living"/ or activity of daily living.mp. OR functional limitation.mp.) NOT body mass index.m_titl.
4	(valid*.mp. OR validation.mp. or Validation Study/ OR reliab*.mp. OR "Reproducibility of Results"/ or reproducib*.mp. OR psychometrics*.mp. or Psychometrics/ OR Psychometrics/ or psychometric properties.mp. OR develop*.mp. OR internal consistency.mp.) NOT "randomi*".m_titl.
5	1 AND 2 AND 3 AND 4.
6	5 Limited to English.

References: #, number.

### Stage three: selection of articles

The articles retrieved in the systematic search were exported to a literature management program (EndNote 20, The EndNote Team, 2013, Philadelphia, PA, Clarivate) and duplicates removed. Articles were then exported to a systematic review software (Covidence, https://www.covidence.org/) to continue the selection process. The screening process was guided by Polanin et al.^34^ Each article was independently screened by two reviewers (the primary investigator [AP] screened all the articles, and four secondary reviewers [FV, PP, RC, and SS] screened one-quarter of the articles each). When two reviewers disagreed on whether an article should be included, they initially attempted to resolve the disagreement via consensus. If consensus could not be achieved, a third reviewer (BD) resolved discrepancies as necessary. A screening tool was developed and used by the review team to help with the screening task. The review team was trained to use the screening tool and Covidence. Pilot screening was conducted prior to commencing the title and abstract screening phase to ensure a shared understanding of the inclusion/exclusion criteria. After two rounds of pilot testing, each reviewer pair achieved >89% agreement. Full-text was retrieved for articles that appeared to meet the inclusion criteria (and no exclusion criteria). Full-text review was done by two reviewers working independently from each other (AP and FV or PP or RC or SS). Finally, we performed a search of reference lists of included studies to identify additional studies of relevance (i.e., citation searching).

### Stage four: data extraction

Data extraction, analysis, and presentation of results were guided by recommendations made by Pollock et al.^35^ Extracted data included relevant publication information (e.g., title, author, year, and journal), study design, number of participants, participants characteristics (e.g., age, sex, MSK condition), outcome measure tested, included physical function(s), measurement property assessed, and test information (e.g., test instructions, scoring system, required equipment, and space). The data extraction sheet was developed and reviewed by the whole review team. Data extraction was done by the primary investigator (AP) and reviewed by the wider team.

## Synthesis of results

Ordinal variables were summarized using counts and percentages. Continuous data were reported as mean and standard deviation (SD) or median and interquartile range (IQR), as appropriate. Rankings of the most assessed physical functions for each of the included populations were developed. We linked each extracted physical function to an ICF code.^10^ The ICF framework provides a standard language and a conceptual basis for the definition and measurement of health and disability. It is composed of 4 concepts: 1) body functions, 2) body structures, 3) activity and participation, and 4) environmental factors.^33^ Each concept can be subclassified in different subcategories. The linking steps were based on work from Cieza et al.^36^ linking rules with slight modifications. Firstly, the primary investigator (AP) established the link between the extracted physical function and the ICF code. In a second step, three investigators (BD, WT, and RS) reviewed this coding and reached consensus. Codes on which no consensus was reached were discussed until agreement was reached. For those physical functions allocated in “other, specified” ICF codes (e.g., d4108 “Changing basic body position, other specified”) inductive thematic analysis was used to create subgroups within these codes. Measurement properties were extracted and grouped according to the COSMIN taxonomy into the four categories: validity (content validity, construct validity, and criterion validity), reliability (test-retest reliability, internal consistency, and measurement error), responsiveness and interpretability (floor and ceiling effects, minimal detectable change, minimum clinical important difference, and substantial clinical benefit).^32^

## Results

**Fig. 1:**
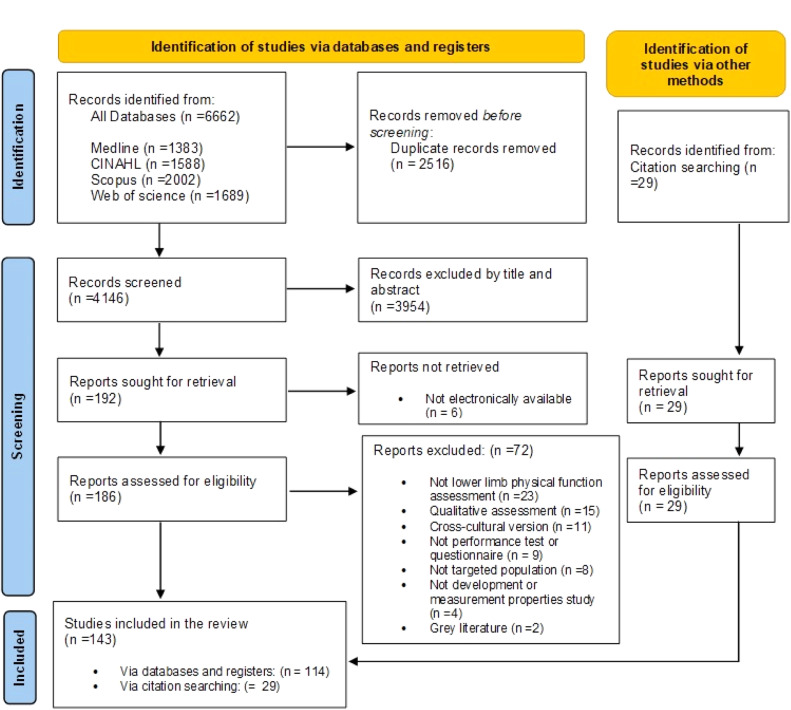
PRISMA flow diagram for the Scoping review.

After removal of the duplicates, we screened 4173 titles and abstracts and reviewed 215 full-text articles (186 identified from databases and 29 from citation searching). After excluding 72 articles, data were extracted from 143 articles (Fig. 1). Eighty-eight studies included participants with knee OA, 49 included participants with ACL injuries, and 19 included participants with PFP (7 studies included two populations and 3 studies included all three populations). Compared to ACL and PFP studies, knee OA studies included larger (median number of participants 93 vs. 73 and 50, respectively) and older (mean age 62 years old vs. 28 and 29) populations. Knee OA and PFP studies included a greater proportion of females (63% and 54%, respectively) than ACL studies (42%) (Supplementary material 2 – Tables A, B, and C). Reliability was the most reported measurement property in knee OA (81%) and PFP (73%) studies, whereas validity was the most reported in ACL studies (71%).

710 physical functions were extracted from 93 different measures and mapped to 50 single ICF codes. The 15 most assessed physical functions are presented in Table 2. An additional 56 physical functions can be found in an extended table in the Supplementary material 3. These other 56 physical functions were assessed between 1 and 12 times, with 30 of these being assessed only once. Climbing stairs was the most assessed physical function in measures for knee OA and PFP, whereas jumping was the most assessed in measures for ACL injuries.

**Table 2:** Ranking of the top 15 physical functions assessed by SROMs and PBOMs for populations with knee OA, ACL injuries, and PFP.

Physical function^a^	Ranking (most often assessed =1)				ICF code	Total times assessed	Physical functions included in n (%) measures			
							Total (N=93)	SROMs (N=37)	PBOMs (N=53)	Mixed (N=3)
	All	Knee OA	ACL	PFP						
Climbing stairs	1	1	2	1	d4551	93	42 (45)	31 (84)	9 (17)	2 (67)
Standing up from sitting	2	2	5	4	d4104	68	40 (43)	25 (68)	13 (25)	2 (67)
Walking short distances	3	3	6^b^	3	d4500	58	40 (43)	21 (58)	17 (32)	2 (67)
Jumping	4	20^b^	1	7 b	d4553	45	28 (30)	14 (39)	12 (23)	2 (67)
Squatting	5	5^b^	4	2	d4101	40	34 (37)	23 (62)	8 (15)	3 (100)
Sitting down from standing	7	4	9^b^	5^b^	d4103	35	27 (29)	18 (49)	8 (15)	1 (33)
Running	6	13	3	5^b^	d4552	32	20 (22)	16 (44)	2 (4)	2 (67)
Maintaining a standing position	8	5^b^	6^b^	10^b^	d4154	26	16 (17)	13 (36)	2 (4)	1 (33)
Getting in/out of a vehicle	9	5^b^	17^b^	10^b^	d4108^c^	21	14 (15)	12 (33)	1 (2)	1 (33)
Kneeling	10	10^b^	8	9	d4102	19	16 (17)	15 (42)	0 (0)	1 (33)
Bending	11^b^	9	11^b^	–	d4105	17	15 (16)	12 (33)	2 (4)	1 (33)
Putting on footwear	11^b^	8	17^b^	16^b^	d5402	17	15 (16)	14 (39)	0 (0)	1 (33)
Turning, twisting, pivoting	13	10^b^	13^b^	16^b^	d4108^c^	16	15 (16)	6 (17)	9 (17)	0 (0)
Maintaining a sitting position	14	10^b^	25^b^	7^b^	d4153	15	8 (9)	7 (19)	1 (2)	0 (0)
Walking long distances	15	15^b^	13^b^	10^b^	d4501	13	7 (8)	5 (14)	1 (2)	1 (33)

Abbreviations: %, percentage; ACL, anterior cruciate ligament; ICF, International Classification of Functioning, Disability and Health; N, number of measures; OA, osteoarthritis; PBOMs, performance-based outcome measures; PF, physical function; PFP, patellofemoral pain; SROMs, self-reported outcome measures.

a Order based on the column “All”.

b Denotes equal ranking.

c Thematic subclassification.

**Fig. 2:**
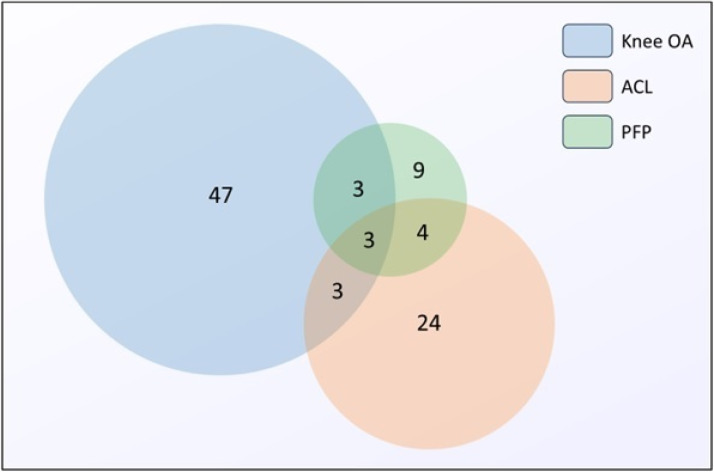
Number of measures across the different populations. Each circle represents a specific knee population. The size of the circle corresponds to the number of measures for each population. Shared areas represent measures common for more than one population (i.e., knee OA has 56 measures in total). Abbreviations: ACL, anterior cruciate ligament; OA, osteoarthritis; PFP, patellofemoral pain.

Of the 93 extracted measures, 53 were PBOMs, 37 were SROMs, and 3 were mixed measures (i.e., include both self-reported items and performance tests). Fifty-six measures assessed knee OA (31 PBOMs, 24 SROMs, and 1 mixed measure), 34 ACL injuries (18 PBOMs, 14 SROMs, and 2 mixed measures), and 19 PFP (7 PBOMs, 11 SROMs, and 1 mixed measure). Fig. 2 shows the number of measures for each or multiple populations.

### ICF coding

The majority of the extracted physical functions were linked to an existing ICF code (Open access Excel file link: https://shorturl.at/vxHch). Ten items were unable to be linked to a single ICF code because these referred to more than one activity. Table 3 shows the thematic classification developed for the ‘other specified’ ICF codes which resulted in 12 new codes.

**Table 3:** Thematic subclassification of “other, specified” ICF codes.

ICF code	Code description (N)	Thematic classification (N)
d4108	“Changing basic body position, other specified” (71)	“Getting in/out of a vehicle” (21) “Changing basic position in bed” (7) “Twisting, turning, pivoting” (16) “Getting on/off of a toilet” (11) “Getting in/out of a bath/tub” (12) “Others”^a^ (5)
d4158	“Maintaining a body position, other specified” (7)	“Standing on one leg” (4) “Others”^a^ (3)
d4508	“Walking, other specified” (8)	“Different types of walking” (4) “Walking in different situations” (4)
d4558	“Moving around, other specified” (25)	“Movements during running (cuts, turns” (9) “Accelerating, decelerating while running” (8) “Others”^a^ (8)
d6408	“Doing housework, other specified” (19)	“Light housework” (9) “Heavy housework” (10)

Abbreviations: ICF, International Classification of Functioning, Disability and Health; N, number of times assessed.

a “Others” represent physical functions that were not able to be assigned to a common theme. These physical functions were considered individually for the development of the ranking.

### Characteristics of the PBOMs

The median number of physical functions assessed by PBOMs was 1 (IQR 1–2; range 1 - 12) with 31 tests (58%) assessing a single physical function. The three most assessed physical functions were “Walking short distances”, “Jumping” and “Standing up from sitting” (Supplementary material 4). The time required to complete a task, the total number of repetitions, and the maximum distance achieved were the three most common methods of measurement, included in 45%, 26%, and 19% of all PBOMs, respectively (Supplementary material 5). Bilateral activities (e.g., squatting) were assessed in 53% of the PBOMs, whereas 43% assessed unilateral activities (e.g., hopping). Only two measures assessed a combination of both bilateral and unilateral activities.

### Characteristics of the SROMs

The median number of physical functions assessed by SROMs was 9 (IQR 7–17; range 1 - 124). The 3 most assessed physical functions were “Climbing stairs”, “Walking short distances”, and “Standing up from sitting” (Supplementary material 6). Most SROMs (70%) used a 5-point Likert scale as the scoring method (Supplementary material 7). We found large variability regarding the description of items assessing the same physical function across different SROMs (Open access Excel file link: https://shorturl.at/vxHch). Virtually all items included in SROMs explored bilateral functions (e.g., walking or squatting). When unilateral functions were included (like hopping), the limb used was not specified.

## Discussion

This scoping review explored the content of measures used to evaluate lower limb physical function in people with knee MSK conditions. We identified 93 different measures that assessed 710 discrete physical functions that were linked to 50 ICF codes. The majority of the measures were developed/assessed in people with knee OA. SROMs assessed a much larger spectrum of physical functions than PBOMs. Climbing stairs and standing up from sitting were the most commonly assessed functions in SROMs, whereas walking short distances and jumping were the most commonly assessed functions in PBOMs. Measures for people with ACL injuries assessed more dynamic and challenging functions than those for people with knee OA and PFP, which focused in lower difficulty daily tasks.

Overall, the five most assessed physical functions were climbing stairs, standing up from sitting, walking short distances, jumping, and squatting. However, we found slight differences in the rankings within each clinical population. Weight-bearing activities (e.g., climbing stairs, standing up from sitting) were the most assessed physical activities in measures for people with knee OA and PFP. Jumping was the most assessed physical function in measures for people with ACL injuries. The most commonly assessed physical functions for each population reflect key symptoms and impairments (commonly impacted activities) or the reason for the test (such as assessing readiness to return to sport in people with ACL injuries).^37^, ^38^, ^39^, ^40^, ^41^, ^42^, ^43^ However, this focus on conditions’ key characteristics might limit assessment to a narrower level of function and reduce applicability to diverse populations or symptom states. Challenging and high-impact activities such as jumping and running were highly assessed within measures for people with ACL injuries (ranked 1st and 3rd, respectively), moderately assessed in measures for PFP (ranked 7th and 5th, respectively), but rarely assessed (ranked 20th and 13th, respectively) in measures for knee OA. This may be due to people with ACL injuries and PFP commonly being young and physically active. However, this is evidence of a gap in the assessment of demanding activities in people with knee OA. Assessing these demanding activities could be important for the detection of people with early symptoms of knee OA who might also be young and physically active or in need of monitoring functional change over time.^38^^,^^44^^,^^45^

There was a large contrast in the number of physical functions assessed by self-reported and performance-based measures. PBOMs assessed fewer physical functions than SROMs (median of 1 vs. 9). This means that multiple PBOMs may be necessary to have complete objective evaluation of a person's physical ability, however, this would be difficult to achieve given the different scoring methods used. Unilateral activities were more commonly assessed in PBOMs than in SROMs. This suggests that performance tests might be better suited to detect differences between injured and non-injured limbs.

Most physical functions could be linked to an ICF code. However, some issues were identified through this process. We identified several items in SROMs that attempted to measure multiple physical functions (e.g., “bending, kneeling, or stooping” or “vacuuming or yard work”). These multiple function items are likely to generate interpretation and response variability affecting measurement quality. Some items were vague in their description (e.g., “stairs”, “sit”, “standing”), similarly allowing broad interpretation. The ICF lacked codes for some activities that were frequently assessed in measures and represent common activities of daily living such as ‘getting in/out of vehicles’; ‘turning, twisting, or pivoting’ and ‘getting in/out of a bath’. Likewise, challenging activities such as ‘cuts/sharp turns’ or ‘accelerating/decelerating while running’ lacked specific ICF codes. Some of these issues have been reported previously and may represent an opportunity to improve the ICF.^46^^,^^47^

Key gaps we identified in available measures to assess knee physical function are the inclusion of very few physical functions in PBOMs, knee OA measures generally not including challenging activities, and high variability among the wording and specificity of items in SROMs. Addressing these gaps when selecting or drafting items/activities can help clinicians and researchers develop better and more comprehensive measures. Based on the findings of this research, we suggest: 1) including several physical functions across different levels of difficulty that can be scored using the same method in a single PBOM to enable generation of a single score that reflects an individual's overall lower limb physical function; 2) avoiding use of multi-activity items in SROMs given these might be interpreted differently by different respondents; 3) developing SROMs that include unilateral functions; 4) including more challenging activities in scales for OA to better reflect the diverse ability in this group; and 5) being as specific as possible when drafting questionnaire items. Items that are vague or not detailed enough may be hard to interpret, and therefore, difficult to rate consistently. For example, there is a clear difference between “climbing stairs” and “climbing up and down 3 flights of stairs indoors, using a handrail”. Even though both items assess climbing stairs, the latter describes a more precise context to rate.

### Clinical implications

When choosing an outcome measure, clinicians should consider someone's current and potential future functional states. Clinicians may need to be cautious when using PBOMs as measures of overall physical function given that most of these include discrete activities and cannot be combined or compared with other measures of function. When using SROMs to capture impairments related to an injured lower limb, clinicians should consider how they instruct patients as nearly all included tasks require bilateral performance.

### Limitations

The following limitations should be considered when interpreting study results. This review included studies that assessed measurement properties, but did not assess these properties or conduct quality assessment. As a scoping review, the goal was to identify physical functions assessed rather than identify the psychometric properties of individual physical functions or the different ways of assessing these. Given the large number of ways many physical functions were assessed, this may be a useful area for future work. The dataset volume was large, and some minor errors may have arisen from a single researcher undertaking all data extraction, but this also ensured consistency. All extracted data are available in the open access file, allowing cross-checking by others. The exclusion of non-English articles and those published in the grey literature may have missed some measures, however, we retrieved a large number of measures, including those developed in non-English speaking countries that were published in English language journals. We included measures assessing three of the most common knee conditions. These cover acute, persistent, and long-term symptom states and affect people across the life course. However, these findings may not generalise to all other knee conditions as these may be better assessed by measures not mentioned in this review.

## Conclusion

Measures to assess lower limb physical functions include a wide variety of activities. Climbing stairs is the most assessed physical function in outcome measures for knee OA and PFP populations, whereas jumping is the most assessed physical function in outcome measures for the ACL injury population. Challenging and more difficult activities are rarely assessed in the knee OA outcome measures. SROMs generally assess a broader range of bilateral physical functions, whereas PBOMs focus on discrete physical activities (bilateral or unilateral) and are difficult to combine due to different scoring systems. Current physical function outcome measures are not well suited to assess performance in OA populations with mild or diverse levels of impairment.

## Data sharing

The review protocol, as well as the complete dataset are available on Open Science Framework, a public open-access repository.

## Declaration of competing interest

The authors declare that they have no known personal relationships that could have appeared to influence the work reported in this paper.

## Appendix Supplementary materials

Image, application 1
