# Supplementary material for: Physical functions assessed by lower limb performance-based and self-reported outcome measures for knee musculoskeletal conditions: A scoping review
Source: Braz J Phys Ther. 2024 Dec 12;29(1):101166. doi: 10.1016/j.bjpt.2024.101166 (PMC11698925; doi:10.1016/j.bjpt.2024.101166)
Supplement: Supplementary file 1 [file mmc1.pdf]

Supplementary material 1- Search strategy for the scoping review

Search date: 07/02/23

Medline

SET #		Results
1	(knee osteoarthritis.mp. or Osteoarthritis, Knee/ OR knee OA.mp. OR osteoarthritis of the knee.mp. OR knee arthritis.mp. OR arthritis of the knee.mp. OR Anterior Cruciate Ligament Injuries/ or Anterior Cruciate Ligament/ or anterior cruciate ligament.mp. OR Anterior Cruciate Ligament Reconstruction/ or ACL.mp. OR Patellofemoral Pain Syndrome/ or patellofemoral knee pain syndrome.mp. OR patellofemoral pain syndrome.mp. OR patellofemoral pain.mp. OR PFPS.mp.) NOT (knee replacement.m_titl. OR joint replacement.m_titl. OR "arthroplast*".m_titl. OR meniscectomy.m_titl. OR "surg*".m_titl. OR "arthroscop*".m_titl. OR "corticoid*".m_titl.)	50293
2	outcome measure.mp. or Outcome Assessment, Health Care/ OR questionnaire.mp. or "Surveys and Questionnaires"/ OR test*.mp. OR performance based*.mp. OR performance measure.mp. OR performance instrument.mp. OR performance scale.mp. OR performance index.mp. OR Patient Reported Outcome Measures/ or patient reported*.mp. OR self report*.mp. OR observation measure.mp.	5557151
3	(performance.mp. or Athletic Performance/ OR Disability Evaluation/ or "International Classification of Functioning, Disability and Health"/ or disability.mp. OR "Activities of Daily Living"/ or physical function.mp. OR functionality.mp. OR function.mp. OR return to sport.mp. or Return to Sport/ OR physical activity.mp. or Exercise/ OR functional activity.mp. OR deficit.mp. OR "Activities of Daily Living"/ or activity of daily living.mp. OR functional limitation.mp.) NOT body mass index.m_titl.	4327372
4	(valid*.mp. OR validation.mp. or Validation Study/ OR reliab*.mp. OR "Reproducibility of Results"/ or reproducib*.mp. OR psychometrics*.mp. or Psychometrics/ OR Psychometrics/ or psychometric properties.mp. OR develop*.mp. OR internal consistency.mp.) NOT "randomi*".m_titl	7066343

5	1 AND 2 AND 3 AND 4	1452
6	5 Limited to English	1383

CINAHL

SET #		Results
1	((MH "Osteoarthritis, Knee") OR "knee osteoarthritis" OR "osteoarthritis of the knee" OR "osteoarthritis of knee" OR "knee arthritis" OR "arthritis of knee" OR "knee oa" OR (MH "Anterior Cruciate Ligament") OR "anterior cruciate ligament" OR (MH "Anterior Cruciate Ligament Injuries") OR (MH "Anterior Cruciate Ligament Reconstruction") OR "acl" OR "acl reconstruction" OR (MH "Patellofemoral Pain Syndrome") OR "patellofemoral pain syndrome" OR "patellofemoral pain" OR "PFPS") NOT (TI knee replacement OR TI arthroplast* OR TI meniscectomy OR TI surg* OR TI arthroscop* OR TI corticoid*)	46,134
2	(MH "Outcome Assessment") OR "outcome measure" OR "questionnaire" OR (MH "Questionnaires") OR "performance test" OR (MH "Performance Measurement Systems") OR (MH "Exercise Test") OR "performance measure" OR "performance based" OR "performance instrument" OR "performance index" OR "performance assessment" OR (MH "Scales") OR "performance scale" OR "performance index" OR "performance assessment" OR (MH "Self Report") OR "self report*" OR (MH "Patient-Reported Outcomes") OR "patient reported*”	1,891,471
3	((MH "Disability Evaluation") OR "disability" OR "physical function" OR (MH "Functional Assessment") OR "functionality" OR (MH "Athletic Performance") OR "deficit" OR (MH "Sports Re-Entry") OR "return to sport" OR (MH "Physical Activity") OR "physical activity" OR (MH "Activities of Daily Living") OR "functional activity" OR "functional limitation" OR "function") NOT TI body mass index	836,240
4	(MH "Validity") OR "validity" OR (MH "Predictive Validity") OR (MH "Criterion-Related Validity") OR (MH "Reliability and Validity") OR (MH "Internal Validity") OR (MH "Face Validity") OR (MH "External Validity") OR (MH "Discriminant Validity") OR (MH "Consensual Validity") OR (MH	1,569,758

	"Construct Validity") OR (MH "Content Validity") OR "validation" OR (MH "Validation Studies") OR (MH "Reliability") OR "reliability" OR (MH "Interrater Reliability") OR (MH "Reliability and Validity") OR (MH "Test-Retest Reliability") OR (MH "Intrarater Reliability") OR (MH "Reproducibility of Results") OR "reproducib*" OR (MH "Psychometrics") OR "psychometrics*" OR "develop*" OR (MH "Internal Consistency") OR "internal consistency") NOT TI randomi*	
5	1 AND 2 AND 3 AND 4	1,674
6	5 Limited to English	1,588

Web of science

SET#		Results
1	ACL or "anterior cruciate ligament" or "knee osteoarthritis" or "knee OA" or "osteoarthritis of the knee" or "knee arthritis" or "arthritis of the knee" or "patellofemoral pain" or "PFPS"	69,809
2	"outcome measure" or test or questionnaire or "performance based" or "performance measure" or "performance instrument" or "performance index" or "performance scale" "patient reported" or "self report*" or "observation measure"	7,771,454
3	performance or disability or "physical function" or functionality or function or "return to sport" or "physical activity" or deficit or "functional activity" or "activities of daily living" or "functional limitation"	11,707,622
4	validation or reliability or validity or psychometrics or "psychometric testing" or "psychometric properties" or development or develop or reproducib* or "internal consistency"	11,950,299
5	TITLE: "knee replacement" OR "arthroplast*" OR meniscectomy OR surg* OR "body mass index" OR randomi* OR "systematic review*" OR "meta-analysis" OR arthroscop* OR corticosteroid*	1,487,770
6	1 NOT 5	56,810
7	2 NOT 5	7,552,014
8	3 NOT 5	11,486,715

9	4 NOT 5	11,736,601
10	6 AND 7 AND 8 AND 9	1802
11	10 Limited to English	1689

Scopus

SET#		Results
1	ACL or "anterior cruciate ligament" or "knee osteoarthritis" or "knee OA" or "osteoarthritis of the knee" or "knee arthritis" or "arthritis of the knee" or "patellofemoral pain" or "PFPS"	89,629
2	"outcome measure" or test or questionnaire or "performance based" or "performance measure" or "performance instrument" or "performance index" or "performance scale" or "patient reported" or "self report*" or "observation measure"	9,067,659
3	performance or disability or "physical function" or functionality or function or "return to sport" or "physical activity" or deficit or "functional activity" or "activities of daily living" or "functional limitation"	15,409,341
4	validation or reliability or validity or psychometrics or "psychometric testing" or "psychometric properties" or development or develop or reproducib* or "internal consistency"	11,986,420
5	TITLE: "knee replacement" OR "arthroplast*" OR meniscectomy OR surg* OR "body mass index" OR randomi* OR "systematic review*" OR "meta-analysis" OR arthroscop* OR corticosteroid*	1,595,625
6	1 NOT 5	69,171
7	2 NOT 5	8,768,136
8	3 NOT 5	15,141,458
9	4 NOT 5	11,816,502
10	6 AND 7 AND 8 AND 9	2,097
11	10 Limited to English	2,002

Supplementary material 2- TABLE A. Characteristics of the articles including participants with knee osteoarthritis (N=88).

Author (Year)	Number of participants	Mean age (SD)	Duration of the symptoms (months)	Kellgren and Lawrence Grade	Female %	Study design	Outcome measure	Measurement properties
Abbott (2018)[1]	206	66.6 (9.5)	30	-	55	Development, longitudinal	Shortened version of the Western Ontario and McMaster Universities Osteoarthritis Index (ShortMAC)	SEM, MDC, MCID,
Akai (2005)[2]	First cohort: 150<br><br>Second cohort: 84	First cohort: 72.7 (9.3)<br><br>Second cohort: 73.4 (7.7)	-	-	First cohort: 85<br><br>Second cohort: 80	Development, longitudinal	Japanese Knee Osteoarthritis Measure	Content validity, construct validity, reliability
Akinpelu (2007) [3]	49	58.3 (13.0)	-	-	59	Development, longitudinal	Ibadan Knee/Hip Osteoarthritis Outcome Measure (IKHOAM)	Content validity, construct validity,
Akinpelu (2011)[4]	97	59.2 (11.5)	38	-	81	Longitudinal	IKHOAM	SEM, MCID
Alghadir (2015)[5]	65	54.3 (10.1)	-	I: 58%<br>II: 19%<br>III: 23%	61	Cross-sectional	Time up and go test	Reliability, SEM, MDC
Almeida (2021)[6]	82	64.2 (9.2)	-	-	82	Longitudinal	Step up and down test	Construct validity, reliability, responsiveness
Almeida (2022)[7]	41	56.4 (7.6)	-	II: 29%<br>III: 71%	85	Cross-sectional	2-minute step test	Construct validity, SEM, reliability, MDC

Author (Year)	Number of participants	Mean age (SD)	Duration of the symptoms (months)	Kellgren and Lawrence Grade	Female %	Study design	Outcome measure	Measurement properties
Alotaibi (2023)[8]	73	58.1 (7.1)	56		59	Development, cross-sectional	Arabic Knee Osteoarthritis Outcome Measure	Construct validity, content validity, reliability
Angst (2001)[9]	223	65.1 (10.0)	-	-	75	Longitudinal	Western Ontario and McMaster Universities Arthritis (WOMAC)	Responsiveness
Ateef (2016)[10]	80	50.7 (15.1)	-	0: 11%<br>I: 16%<br>II: 11%<br>III: 27%<br>IV: 35%	64	Cross-sectional	6-minute walk test	Construct validity, reliability
Beirer (2015)[12]	15	47 (18.0)	-	-	49	Development, longitudinal	Munich Knee Questionnaire	Content validity, construct validity, reliability, responsiveness, floor and ceiling effects
Bellamy (1986)[13]	100	61.1 (27-93)a	120	-	63	Development	WOMAC	Content validity
Bellamy (1988)[14]	57	66.5 (55-82)a	108	-	54	Longitudinal	WOMAC	Construct validity, reliability, responsiveness
Bellamy (2011)[15]	38	68.5 (8.7)	108	III: 43.6%<br>IV: 56.4%	61	Longitudinal	WOMAC	Criterion validity, reliability, responsiveness
Binkley (1999)[18]	9	44 (16.2)	-	-	58	Development, longitudinal	Lower extremity functional scale (LEFS)	Content validity, construct validity, reliability, SEM, MDC, MCID

Author (Year)	Number of participants	Mean age (SD)	Duration of the symptoms (months)	Kellgren and Lawrence Grade	Female %	Study design	Outcome measure	Measurement properties
Brazier (1999)[21]	109	71 (47-87)a	-	-	-	Longitudinal	WOMAC	Construct validity, internal consistency, responsiveness
Chang (2018)[25]	120	65.1 (7.4)	-	-	79	Longitudinal	Osteoarthritis Function Computerized Adaptive Test (OA–FUNCTION-CAT)	Responsiveness
Chaudhary (2021)[26]	82	60.4 (6.6)	-	> I: 100%	67	Cross-sectional	2-minute walk test	SEM, MDC, reliability
Collins (2008)[29]	105	64.5 (8.4)	120	II: 56%<br>III: 44%	11	Cross-sectional	Walking impairment questionnaire	Construct validity, reliability
Conaghan (2007)[33]	224	61 (26-90)a	-	-	61	Cross-sectional	Oxford knee score	Construct validity
Davey (2003)[24]	21	69.5 (7.2)	-	-	33	Cross-sectional	8-foot walking speed<br>Stair-climb test<br>WOMAC	SEM, reliability
Dobson (2017)[38]	51	64.5 (6.2)	84	-	47	Cross-sectional	30-second chair test<br>40-meter fast-paced walk test<br>Stair-climb test<br>Time up and go test<br>6-minute walk test	Reliability, SEM, MDC
Driban (2015)[39]	204	60.2 (10.5)	60	>I: 92%	70	Cross-sectional	Patient-Reported Outcomes Measurement Information System (PROMIS) Short Form Physical Function	Construct validity, floor and ceiling effects

Author (Year)	Number of participants	Mean age (SD)	Duration of the symptoms (months)	Kellgren and Lawrence Grade	Female %	Study design	Outcome measure	Measurement properties
Faucher (2003)[43]	88	67.1 (10.2)	107	Mean 3.2<br>Range 2-4	67	Cross-sectional	Lequesne index	Construct validity, reliability
Franchignoni (2012)[44]	697	72.1 (10.5)	-	-	72	Cross-sectional	Lequesne index	Construct validity, reliability
French (2011)[45]	39	65.3 (6.9)	-	-	82	Longitudinal	WOMAC<br>Lequesne index<br>Time up and go test<br>6-minute walk test<br>Timed stand test	Responsiveness
Gentelle-Bonnassies (2000)[47]	80	62 (12.0)	60	0-II: 50%<br>III-IV: 50%	70	Longitudinal	Lequesne index<br>WOMAC	Responsiveness
Gill (2022)[48]	93	61.3 (8.5)	38	II: 100%	59	Cross-sectional	30-second chair test	Reliability, SEM, MDC
Harris (2013)[51]	161,973	69 (18-97)a	-	-	56	Cross-sectional	Oxford knee score	Construct validity
Harris (2013)[75]	134	59 (11.0)	-	0:2%<br>I: 8%<br>II: 43%<br>III: 16%<br>IV: 4%	50	Longitudinal	Oxford knee score	Construct validity, reliability, SEM, responsiveness, MDC, MCID
Higgins (2007)[52]	1,517	37.5 (14.9)	-	-	42	Cross-sectional	International Knee Documentation Committee (IKDC)	Construct validity, reliability
Hoglund (2019)[54]	20	58.3 (8.1)	-	-	100	Cross-sectional	30-second fast-paced walk test	Construct validity, reliability, SEM, MDC
Holm (2021)[55]	40	68.8 (8.3)	-	>I: 100%	55	Cross-sectional	Stair-climb test<br>40-meter fast-paced walk test	Reliability

Author (Year)	Number of participants	Mean age (SD)	Duration of the symptoms (months)	Kellgren and Lawrence Grade	Female %	Study design	Outcome measure	Measurement properties
							30-second chair test	
Howe (1995)[58]	27	73b	-	-	50	Cross-sectional	Time stand test<br>10-meter walk test	Reliability
Iijima (2019)[59]	59	59.1 (6.1)	1	0: 39%<br>I: 46%<br>II: 15%	73	Cross-sectional	Stair-climb test	Construct validity, reliability, SEM
Irrgang (1998)[63]	397	33.3 (12-76)a	-	-	39	Development, longitudinal	Knee Outcome Survey Activities of Daily Living Scale (KOS)	Content validity, construct validity, reliability, responsiveness
Irrgang (2001)[61]	92	37.5 (16.2)	-	-	47	Development, cross-sectional	IKDC	Content validity, construct validity, reliability
Irrgang (2006)[62]	76	40.5 (16.7)	-	-	53	Longitudinal	IKDC	Responsiveness
Jette (2009)[66]	328	61.8 (15.1)	-	-	64	Development, cross-sectional	OA–FUNCTION-CAT	Content validity, construct validity, reliability, floor and ceiling effects.
Kanko (2019)[67]	74	57.7 (8.8)	-	-	45	Longitudinal	Star excursion balance test	Construct validity, reliability, SEM, MDC, responsiveness
Kersten (2010)[68]	221	66.8 (8.3)	-	-	58	Longitudinal	WOMAC 3.0	Construct validity, responsiveness
Kim (2013)[69]	634	66 (8.0)	-	-	89	Development, longitudinal	Korean knee score	Content validity, construct validity, reliability, responsiveness, floor and ceiling effect

Author (Year)	Number of participants	Mean age (SD)	Duration of the symptoms (months)	Kellgren and Lawrence Grade	Female %	Study design	Outcome measure	Measurement properties
Klokker (2015)[70]	20	64 (6.6)	-	Mean: 2.8<br>Range: 2-4	70	Cross-sectional	Dynamic weight-bearing assessment of pain	Reliability, SEM, MDC
Klokker (2016)[71]	100	63.7 (8.8)	-	Mean: 2.8<br>Range: 2-4	62	Longitudinal	Dynamic weight-bearing assessment of pain	Construct validity, responsiveness, MCID
Lee (2022)[78]	30	63.2 (6.5)	>12: 76%	0: 48%<br>I: 27%<br>II: 25%	90	Cross-sectional	40-meter fast-paced walk test<br>Time up and go test<br>30-second chair test<br>6-minute walk test<br>Stair-climb test	Construct validity
Lequesne (1987)[79]	27	-	-	-	-	Development, cross-sectional	Lequesne index	Content validity
Lin (2001)[80]	89	69.4 (5.9)	144	-	88	Cross-sectional	8-foot walking speed<br>Chair stands<br>Ascending stairs<br>Descending stairs	Construct validity, reliability, SEM
Lyman (2019)[83]	Phase 1: 187<br>Phase 2: 241	Phase 1: 71 (31-88)a<br>Phase 2: 69 (23-94)	-	0: 1%<br>I: 9%<br>II: 21%<br>III: 33%<br>IV: 36%	Phase 1: 82<br>Phase 2: 75	Development, cross-sectional	Japanese Knee Injury and Osteoarthritis Outcome Score (KOOS)<br>KOOS-Knee flexion domain	Content validity, construct validity,
Mahler (2017)[84]	161	59 (9.0)	>60: 37%	-	61	Longitudinal	KOOS-Physical function shortform (KOOS-PS)<br>Lequesne index<br>LEFS	Responsiveness
Master (2018)[87]	1.925	65.1 (9.1)	-	-	55	Longitudinal	Chair stands test<br>20-meter walk test	Criterion validity

Author (Year)	Number of participants	Mean age (SD)	Duration of the symptoms (months)	Kellgren and Lawrence Grade	Female %	Study design	Outcome measure	Measurement properties
							400-meter walk test	
McCarthy (2004)[88]	214	64.7 (9.8)	111	-	-	Longitudinal	Aggregated locomotor function	Construct validity, reliability, SEM, responsiveness, MDC
McKay (2013)[89]	76	64.5 (16.5)	-	-	56	Longitudinal	Lower limb tasks questionnaire	Construct validity, responsiveness
McNair (2007)[90]	174	37 (15.0)	-	-	-	Development, longitudinal	Lower limb tasks questionnaire	Content validity, construct validity, reliability, SEM, MCID, responsiveness, floor and ceiling effects
Mehta (2019)[91]	44	66.9 (8.1)	19	-	61	Cross-sectional	10-meter walk<br>Time up and go test<br>Short physical performance battery	Construct validity
Mills (2016)[92]	272	-	-	-	69	Longitudinal	KOOS	MCID
Mostafaee (2022)[94]	60	59.9 (6.8)	6	II: 47%<br>III: 53%	82	Longitudinal	30-second chair test<br>40-meter fast-paced walk test<br>Stair-climb test	Responsiveness, MCID
Motyl (2013)[95]	15	61 (7.8)	-	II: 20%<br>III: 80%	53	Cross-sectional	20-meter walk test	Reliability, MDC
Nalbant (2022)[97]	25	62.3 (9.8)	-	IV: 100%	80	Cross-sectional	L-test	Reliability, SEM, MDC
Naylor (2014)[98]	75	67.6 (9.4)	-	-	63	Cross-sectional	Time up and go test<br>6-minute walk test<br>KOOS	SEM, MDC

Author (Year)	Number of participants	Mean age (SD)	Duration of the symptoms (months)	Kellgren and Lawrence Grade	Female %	Study design	Outcome measure	Measurement properties
Öberg (1994)[99]	105	69 (46-91)a	-	III: 100%	82	Development, cross-sectional	System of lower extremity Dysfunction	Content validity, construct validity, reliability
Odole (2013)[100]	-	-	-	-	-	Development	IKHOAM	Content validity
Parveen (2017)[102]	25	50.5 (6.3)	44	Mean: 2.2	64	Cross-sectional	Performance-oriented mobility assessment	Construct validity, reliability, SEM, MDC
Perruccio (2008)[106]	2145	64.3 (26-95)	-	-	58	Development, cross-sectional	KOOS-PS	Construct validity, reliability
Peter (2017)[107]	47	64.7 (8.2)	-	-	80	Cross-sectional	Animated activity questionnaire	Construct validity
Peter (2018)[108]	689	64	-	-	71	Cross-sectional	Animated activity questionnaire	Construct validity, reliability, internal consistency, SEM, MDC
Peter (2019)[109]	49	66.2 (8.8)	-	-	67	Longitudinal	Animated activity questionnaire	Responsiveness, MCID
Piva (2004)[110]	105	62 (9.0)	-	> I	76	Cross-sectional	Get up and go test	Construct validity, reliability, SEM, MDC
Pollard (2009)[112]	482	68.8 (9.9)	-	-	53	Development, cross-sectional	Aberdeen activity limitation scale	Content validity, construct validity, reliability
Pollard (2011)[113]	185	68.9 (9.4)	-	-	54	Cross-sectional	Aberdeen activity limitation scale	Construct validity
Rejeski (1995)[116]	440	68.8 (5.6)	-	-	70	Development, cross-sectional	Physical activities restrictions	Content validity, construct validity, reliability

Author (Year)	Number of participants	Mean age (SD)	Duration of the symptoms (months)	Kellgren and Lawrence Grade	Female %	Study design	Outcome measure	Measurement properties
Sharma (2021)[119]	397	63.8 (8.0)	72	II: 35%<br>III: 31%<br>IV: 34%	54	Longitudinal	Stair-climb test	Construct validity, reliability, SEM, MDC, MCID, responsiveness
Singh (2014)[120]	141	60.8 (11.4)	111	-	43	Longitudinal	KOOS-PS	Reliability, MDC, MCID
Steultjens (1999)[121]	119	68 (8.9)	-	-	78	Cross-sectional	Stand-to-sit time<br>5-meter walking time<br>Stand-to recline time	Construct validity, reliability
Steultjens (2001)[122]	112	67.9 (8.7)	-	-	80	Longitudinal	Stand-to-sit time<br>5-meter walking time<br>Stand-to recline time	Responsiveness
Stratford (2003)[123]	93	63.2 (11.2)	-	-	49	Cross-sectional	40-meter fast-paced walk test<br>Time up and go test<br>Stair-climb test<br>LEFS	Construct validity
Stratford (2006)[125]	96	65 (58-73)b	-	-	51	Cross-sectional	40-meter fast paced walk test<br>Time up and go test<br>Stair-climb test<br>6-minute walk test	Construct validity
Stratford (2014)[124]	377	64.4 (10.5)	-	-	63	Cross-sectional	KOOS Fun-sport	Construct validity, floor and ceiling effects
Suwit (2020)[126]	55	69 (11.0)b	-	-	67	Cross-sectional	40-meter fast-paced walk test<br>Stair-climb test<br>30-second Chair test	Construct validity, reliability, SEM, MDC

Author (Year)	Number of participants	Mean age (SD)	Duration of the symptoms (months)	Kellgren and Lawrence Grade	Female %	Study design	Outcome measure	Measurement properties
Takacs (2014)[127]	25	62.5 (7.4)	-	II: 56%<br>III: 36%<br>IV: 8%	56	Cross-sectional	Community balance and mobility scale	Construct validity, reliability, SEM
Takacs (2017)[128]	131	66.3 (8.5)	-	I: 10%<br>II: 47%<br>III: 28%<br>IV: 15%	61	Cross-sectional	Community balance and mobility scale	Construct validity
Tanimura (2011)[129]	362	72.4 (9.6)	> 12: 85%	-	78	Development, cross-sectional	Difficulties in daily life of patients with knee osteoarthritis scale	Content validity, construct validity, reliability
Terwee (2014)[131]	33	62 (11.0)	-	-	73	Development, cross-sectional	Animated activity questionnaire	Content validity, construct validity
Tolk (2019)[132]	Total cohort: 85<br>Reliability cohort: 30	Total cohort: 69.3 (8.2)<br>Reliability cohort: 67.8 (7.7)	-	-	Total cohort: 57<br>Reliability cohort: 43	Longitudinal	40-meter fast-paced walk test<br>30-second Chair test<br>Stair-climb test	Construct validity, reliability, SEM, MDC, responsiveness
Tubach (2005)[133]	603	67.9 (10.2)	56	II: 18%<br>III: 44%<br>IV: 38%	70	Longitudinal	WOMAC	MCID
Villadsen (2012)[135]	11	68.8 (8.7)	-	-	54	Cross-sectional	20-meter walk test<br>Chair stands<br>30-second unilateral knee bending	Reliability, MDC
Whitehouse (2008)[138]	Validity: 66<br><br>Reliability: 103	-	-	-	-	Cross-sectional	ShortMAC	Criterion validity, reliability

Author (Year)	Number of participants	Mean age (SD)	Duration of the symptoms (months)	Kellgren and Lawrence Grade	Female %	Study design	Outcome measure	Measurement properties
Williams (2012)[140]	168	64.4 (8.7)	-	I: 1%<br>II: 14%<br>III: 49%<br>IV: 36%	65	Longitudinal	KOS Activities of daily living scale<br>LEFS<br>WOMAC	Reliability, SEM, MDC, MCID
Yang (2007)[142]	100	54.9 (34-85)a	-	I-III	37	Longitudinal	ShortMAC	Construct validity, reliability, responsiveness, floor and ceiling effects
Knee OA summary	93 (51-187)b	62.3 (8.3)			63			

Abbreviations: %, percentage; MCID, minimal clinical important difference; MDC, minimal detectable change; OA, osteoarthritis; SD, standard deviation; SEM, standard error of measurement

a: Minimum and maximum (range)
b: Median and interquartile ranges

Supplementary material 2- TABLE B. Characteristics of the articles including participants with ACL injuries (N=49).

Author (Year)	Number of participants	Mean age (SD)	Female %	Study design	Outcome measure	Measurement properties
Barber-Westin (1999)[11]	250	29 (14 - 58)a	41	Longitudinal	Cincinnati knee rating system	Construct validity, reliability, responsiveness, floor and ceiling effects
Beirer (2015)[12]	21	47 (18.0)	49	Development, longitudinal	Munich knee questionnaire	Content validity, construct validity, reliability, responsiveness, floor and ceiling effects
Bengtsson (1996)[16]	7	26.3 (7.8)	-	Cross-sectional	Lysholm knee scoring scale	Reliability
Bjorklund (2006)[20]	59	31 (18-50)a	32	Cross-sectional	Test of athletes with knee injuries	Reliability
Bjorklund (2009)[19]	35	27 (18-50)a	37	Longitudinal	Test of athletes with knee injuries	Construct validity, responsiveness, floor and ceiling effects
Briggs (2009)[22]	1075	37 (18-77)a	42	Longitudinal	Lysholm knee scoring scale	Construct validity, reliability and responsiveness, MDC
Brosky (1999)[23]	15	26 (7.3)	0	Cross-sectional	Single leg hop<br>6-meter time hop<br>Vertical jump	Reliability
Chen (2020)[27]	77	30.4 (7.0)	17	Development, longitudinal	Knee stability in sports/cutting-Pivoting ability scale	Construct validity, reliability, responsiveness, floor and ceiling effects.
Comins (2013)[30]	17	30.6 (23-53)a	71	Development	Knee numeric-entity evaluation score	Content validity

Author (Year)	Number of participants	Mean age (SD)	Female %	Study design	Outcome measure	Measurement properties
Comins (2013)[31]	242	-	43	Cross-sectional	Knee numeric-entity evaluation score	Construct validity
Comins (2018)[32]	166	32 (9.4)	41	Longitudinal	Knee numeric-entity evaluation score<br>IKDC<br>Lysholm knee scoring scale<br>KOOS	Responsiveness
Day (2021)[35]	1126	30.6 (12.6)	50	Cross-sectional	PROMIS mobility	Construct validity, floor and ceiling effects
deFontenay (2022)[36]	35	28.5 (7.5)	43	Longitudinal	IKDC<br>Step-down endurance test<br>Star excursion balance test	Criterion validity
Dobija (2019)[37]	33	36.8 (10.4)	39	Cross-sectional	Star excursion balance test	Construct validity, reliability, SEM, MDC
Duckett (2021)[40]	77	21.9 (7.8)	48	Cross-sectional	IKDC-8	Construct validity, reliability
Fältström (2021)[41]	117	20 (2.0)	100	Longitudinal	Star excursion balance test<br>Single leg hop<br>Side hop	Criterion validity
Fältström (2021)[42]	117	20 (2.0)	100	Longitudinal	KOOS<br>IKDC<br>Star excursion balance test<br>Single leg hop<br>Side hop<br>5-jump test	Criterion validity
Garrison (2015)[46]	40	17.2 (3.8)	50	Longitudinal	Y balance test	Criterion validity
Grindem (2011)[49]	91	29.2 (8.8)	49	Longitudinal	Single leg hop<br>6-meter time hop<br>Crossover hop	Criterion validity

Author (Year)	Number of participants	Mean age (SD)	Female %	Study design	Outcome measure	Measurement properties
					Triple single hop	
Gustavsson (2016)[50]	65	29 (8.0)	40	Development, Cross-sectional	Vertical jump<br>Single leg hop<br>Drop jump followed by a double hop for distance<br>Side hop<br>Square hop	Reliability
Hildebrandt (2015)[53]	28	24.1 (2.5)	54	Development, cross-sectional	Speedy jump	Content validity, reliability
Hooper (2001)[56]	45	31.1 (8.9)	24	Cross-sectional	Hughston clinic questionnaire	Construct validity
Hopper (2002)[57]	19	26.8 (8.4)	32	Cross-sectional	6-meter time hop<br>Crossover hop<br>Stair hop	Reliability
Ingelsrud (2018)[60]	542	29.9 (11.6)	53	Longitudinal	KOOS	MCID
Irrgang (1998)[63]	397	33.3 (12-76)a	39	Development, longitudinal	KOS Activities of Daily Living Scale	Content validity, construct validity, reliability, responsiveness
Irrgang (2001)[61]	129	37.5 (16.2)	47	Development, cross-sectional	IKDC	Content validity, construct validity, reliability
Irrgang (2006)[62]	50	40.5 (16.7)	53	Longitudinal	IKDC	Responsiveness
Jacobs (2018)[64]	1904	-	-	Development, longitudinal	KOOS global	Construct validity, responsiveness, floor and ceiling effects
Jeon (2022)[65]	76	28.6 (12.0)	14	Longitudinal	IKDC<br>Lysholm knee scoring scale	Substantial clinical benefit

Author (Year)	Number of participants	Mean age (SD)	Female %	Study design	Outcome measure	Measurement properties
Kocher (2010)[72]	129	14.6 (2.5)	51	Longitudinal	Pedi-IKDC	Construct validity, reliability, responsiveness, floor and ceiling effects
Kong (2012)[73]	30	23.4 (3.2)	0	Cross-sectional	Single leg hop<br>Shuttle run test<br>Carioca test	Reliability
Kramer (1992)[74]	38	25 (7.0)	42	Cross-sectional	Single leg hop	Reliability
Logerstedt (2012)[81]	120	26 (15-54)a	46	Longitudinal	Single leg hop<br>Crossover hop<br>Triple single hop<br>6-meter time hop	Criterion validity
Marmura (2022)[85]	618	19 (3.0)	51	Cross-sectional	KOOS	Construct validity
Marx (2001)[86]	Reliability: 41<br>Responsiveness: 42<br>Construct validity: 133	Reliability: 32.6 (15-60)a<br>Responsiveness: 30.9 (15-61)a<br>Construct validity: 31.5 (14-65)a	Reliability: 51<br>Responsiveness: 55<br>Construct validity: 48	Longitudinal	KOS Activities of daily living scale<br>Cincinnati knee rating system<br>Lysholm knee scoring scale	Construct validity, reliability, responsiveness
McNair (2007)[90]	174	37 (15.0)	-	Development, longitudinal	Lower limb tasks questionnaire	Content validity, construct validity, reliability, SEM, MCID, responsiveness, floor and ceiling effects
Mostafaee (2021)[93]	54	26.4 (5.1)	0	Longitudinal	Star excursion balance test	Responsiveness
Ortqvist (2012)[101]	34	14 (10-16)a	50	Development	KOOS-child	Content validity

Author (Year)	Number of participants	Mean age (SD)	Female %	Study design	Outcome measure	Measurement properties
Paterno (1996)[103]	13	22.4 (3.2)	23	Cross-sectional	Single leg hop	Reliability
Paterno (2022)[104]	159	17.2 (2.6)	70	Longitudinal	IKDC<br>Single leg hop<br>Triple single hop<br>Crossover hop<br>6-meter time hop	Criterion validity
Patterson (2020)[105]	78	28 (15.0)	38	Longitudinal	Single leg hop<br>Crossover hop<br>Side hop<br>One leg rise	Criterion validity
Ra (2014)[114]	134	27.2 (12-53)a	16	Longitudinal	Lysholm knee scoring scale<br>IKDC	Construct validity, responsiveness, floor and ceiling effects
Reid (2007)[115]	42	25.6 (9.2)	45	Longitudinal	Single leg hop<br>Crossover hop<br>Triple single hop<br>6-meter time hop	Reliability, SEM, MDC, responsiveness
Roos (1998)[117]	21	32 (18-46)a	57	Development, longitudinal	KOOS	Content validity, construct validity, reliability, responsiveness
Tegner (1985)[130]	76	27	27	Development, cross-sectional	Lysholm knee scoring scale	Content validity, construct validity
vanMeer (2013)[134]	Construct validity: 100<br><br>Responsiveness: 50	Construct validity: 26 (18-57)a<br><br>Responsiveness: 28 (18-46)a	Construct validity: 25<br><br>Responsiveness: 24	Longitudinal	KOOS<br>IKDC	Content validity, construct validity, reliability, SEM, MDC, responsiveness, floor and ceiling effects

Author (Year)	Number of participants	Mean age (SD)	Female %	Study design	Outcome measure	Measurement properties
	Reliability: 50	Reliability: 27 (18-48)a	Reliability: 40			
Wellsandt (2018)[137]	76	28.7 (11.3)	35	Longitudinal	Single leg hop<br>6-meter time hop<br>KOS Activities of daily living scale	Criterion validity
Williams (2020)[139]	319	29.1 (9.0)	18	Cross-sectional	IKDC	Construct validity
Woon (2021)[141]	20	26.9 (8.3)	25	Longitudinal	One leg sit to stand	Criterion validity, construct validity,
ACL summary	76 (35-133)b	28 (6.2)	42			

Abbreviations: %, percentage; ACL, anterior cruciate ligament; MCID, minimal clinical important difference; MDC, minimal detectable change; SD, standard deviation; SEM, standard error of measurement

a: Minimum and maximum (range)
b: Median and interquartile ranges

Supplementary material 2- TABLE C. Characteristics of the articles including participants with PFP(N=19).

Author (Year)	Number of participants	Mean age (SD)	Female %	Study design	Outcome measure	Measurement properties
Bengtsson (1996)[16]	9	24.1 (7.0)	-	Cross-sectional	Lysholm knee scoring scale	Reliability
Bennell (2000)[17]	50	23.8 (8.9)	66	Cross-sectional	Anterior knee pain scale<br>Functional index questionnaire<br>Hughston clinic questionnaire	Reliability
Binkley (1999)[18]	6	44 (16.2)	58	Development, longitudinal	LEFS	Content validity, construct validity,

Author (Year)	Number of participants	Mean age (SD)	Female %	Study design	Outcome measure	Measurement properties
						reliability, SEM, MDC, MCID
Chesworth (1989)[28]	18	29 (20-50)a	67	Longitudinal	Functional index questionnaire	Reliability
Crossley (2004)[34]	67	29.2 (7.5)	-	Longitudinal	Anterior knee pain scale<br>Functional index questionnaire	Construct validity, reliability, SEM, responsiveness, MCID
Irrgang (1998)[63]	397	33.3 (12-76)a	39	Development, longitudinal	KOS Activities of daily living scale	Content validity, construct validity, reliability, responsiveness
Irrgang (2001)[61]	93	37.5 (16.2)	47	Development, cross-sectional	IKDC	Content validity, construct validity, reliability
Irrgang (2006)[62]	19	40.5 (16.7)	53	Longitudinal	IKDC	Responsiveness
Kocher (2010)[72]	116	14.6 (2.5)	51	Longitudinal	Pedi-IKDC	Construct validity, reliability, responsiveness, floor and ceiling effects
Kujala (1993)[76]	16	28.5 (26-31)a	100	Development, cross-sectional	Anterior knee pain scale	Content validity, construct validity
Lee (2013)[77]	179	-	-	Development, cross-sectional	Samsung medical center patellofemoral scoring system	Content validity, construct validity, reliability, floor and ceiling effects
Loudon (2002)[82]	29	27.6 (5.3)	65	Cross-sectional	Anteromedial lunge<br>Step-down<br>Bilateral squat<br>Balance and reach	Reliability, SEM

Author (Year)	Number of participants	Mean age (SD)	Female %	Study design	Outcome measure	Measurement properties
Marmura (2022)[85]	618	19 (3.0)	51	Cross-sectional	KOOS	Construct validity
Marx (2001)[86]	Reliability: 41<br>Responsiveness: 42<br>Construct validity: 133	Reliability: 32.6 (15-60)a<br>Responsiveness: 30.9 (15-61)a<br>Construct validity: 31.5 (14-65)a	Reliability: 51<br>Responsiveness: 55<br>Construct validity: 48	Longitudinal	KOS Activities of daily living scale<br>Cincinnati knee rating system<br>Lysholm knee scoring scale	Construct validity, reliability, responsiveness
Myer (2016)[96]	499	14.1 (1.8)	100	Cross-sectional	Anterior Knee Pain Scale 6-item	Construct validity
Piva (2009)[111]	60	29.9 (9.6)	55	Longitudinal	KOS Activities of Daily Living Scale	Responsiveness, MCID
Schmitt (2010)[118]	Total: 158<br>Paediatric: 46<br>Adolescent: 90<br>Young Adult: 22	-	54	Cross-sectional	IKDC	Construct validity, reliability
Watson (2005)[136]	30	35.2 (9.1)	80	Longitudinal	LEFS<br>Anterior knee pain scale	Reliability, SEM, MDC, responsiveness
Zamboti (2021)[143]	20	25.6 (5.0)	100	Cross-sectional	30-second Chair test<br>Stair climb test<br>6-minute step test	Construct validity, reliability, SEM
PFPS summary	50 (20-133)b	29 (7.6)	55			
Abbreviations: %, percentage; MCID, minimal clinical important difference; MDC, minimal detectable change; PFP, patellofemoral pain; SD, standard deviation; SEM, standard error of measurement<br><br>a: Minimum and maximum (range)<br>b: Median and interquartile ranges						

Supplementary material 3. Ranking of the top 15 physical functions assessed by SROMs and PBOMs for populations with ACL injuries, knee OA, and PFP (Extended version).

Physical function	Ranking (most often assessed =1)				ICF code	Total times assessed	Physical functions included in n (%) measures			
	ALL	Knee OA	ACL	PFPS			Total (N=93)	SROMs (N=37)	PBOMs (N=53)	Mixed (N=3)
Climbing stairs	1	1	2	1	d4551	93	42 (45)	31 (84)	9 (17)	2 (67)
Standing up from sitting	2	2	5	4	d4104	68	40 (43)	25 (68)	13 (25)	2 (67)
Walking short distances	3	3	6[a]	3	d4500	58	40 (43)	21 (58)	17 (32)	2 (67)
Jumping	4	20[a]	1	7[a]	d4553	45	28 (30)	14 (39)	12 (23)	2 (67)
Squatting	5	5[a]	4	2	d4101	40	34 (37)	23 (62)	8 (15)	3 (100)
Sitting down from standing	7	4	9[a]	5[a]	d4103	35	27 (29)	18 (49)	8 (15)	1 (33)
Running	6	13	3	5[a]	d4552	32	20 (22)	16 (44)	2 (4)	2 (67)
Maintaining a standing position	8	5[a]	6[a]	10[a]	d4154	26	16 (17)	13 (36)	2 (4)	1 (33)

Physical function	Ranking (most often assessed =1)				ICF code	Total times assessed	Physical functions included in n (%) measures			
							Total (N=93)	SROMs (N=37)	PBOMs (N=53)	Mixed (N=3)
	ALL	Knee OA	ACL	PFPS						
Getting in/out of a vehicle	9	5[a]	17[a]	10[a]	d4108[b]	21	14 (15)	12 (33)	1 (2)	1 (33)
Kneeling	10	10[a]	8	9	d4102	19	16 (17)	15 (42)	0 (0)	1 (33)
Bending	11[a]	9	11[a]	-	d4105	17	15 (16)	12 (33)	2 (4)	1 (33)
Putting on footwear	11[a]	8	17[a]	16[a]	d5402	17	15 (16)	14 (39)	0 (0)	1 (33)
Turning, twisting, pivoting	13	10[a]	13[a]	16[a]	d4108[b]	16	15 (16)	6 (17)	9 (17)	0 (0)
Maintaining sitting position	14	10[a]	25[a]	7[a]	d4153	15	8 (9)	7 (19)	1 (2)	0 (0)
Walking long distances	15	15[a]	13[a]	10[a]	d4501	13	7 (8)	5 (14)	1 (2)	1 (33)
Getting in/out of a bath/tub	16	14	17[a]	16[a]	d4108[b]	12	9 (10)	9 (24)	0 (0)	0 (0)
Getting in/out of a toilet	17	15[a]	17[a]	-	d4108[b]	11	11 (12)	10 (27)	0 (0)	1 (33)

Physical function	Ranking (most often assessed =1)				ICF code	Total times assessed	Physical functions included in n (%) measures			
							Total (N=93)	SROMs (N=37)	PBOMs (N=53)	Mixed (N=3)
	ALL	Knee OA	ACL	PFPS						
Walking on different surfaces	18[a]	18[a]	17[a]	-	d4502	10	5 (5)	5 (14)	0 (0)	0 (0)
Taking off footwear	18[a]	18[a]	13[a]	-	d5403	10	10 (11)	10 (27)	0 (0)	0 (0)
Doing housework, heavy	18[a]	15[a]	17[a]	16[a]	d6409[b]	10	9 (10)	9 (24)	0 (0)	0 (0)
Doing housework, light	21[a]	20[a]	13[a]	16[a]	d6409[b]	9	9 (10)	9 (24)	0 (0)	0 (0)
Changes of direction while running	21[a]	28[a]	11[a]	10[a]	d4558[b]	9	8 (9)	7 (19)	0 (0)	1 (33)
Accelerating, decelerating	23	35[a]	9[a]	10[a]	d4558[b]	8	8 (9)	8 (22)	0 (0)	0 (0)
Lying down	24[a]	22[a]	25[a]	-	d4100	7	7 (8)	7 (19)	0 (0)	0 (0)
Changing position in bed	24[a]	26[a]	25[a]	10[a]	d4108[b]	7	4 (4)	4 (11)	0 (0)	0 (0)
Putting on clothes	26[a]	22[a]	-	-	d5400	6	4 (4)	3 (8)	0 (0)	1 (33)

Physical function	Ranking (most often assessed =1)				ICF code	Total times assessed	Physical functions included in n (%) measures			
							Total (N=93)	SROMs (N=37)	PBOMs (N=53)	Mixed (N=3)
	ALL	Knee OA	ACL	PFPS						
Lifting	26[a]	22[a]	-	16[a]	d4300	6	3 (3)	3 (8)	0 (0)	0 (0)
Carrying in the hands	26[a]	22[a]	-	-	d4301	6	1 (1)	1 (3)	0 (0)	0 (0)
Washing body parts	29	26[a]	-	-	d5100	5	4 (4)	3 (8)	0 (0)	1 (33)
Maintaining standing on one leg	30[a]	31[a]	25[a]	-	d4158[b]	4	4 (4)	1 (3)	2 (4)	1 (33)
Carrying heavy objects	30[a]	35[a]	25[a]	16[a]	d449	4	4 (4)	3 (8)	1 (2)	0 (0)
Shopping	30[a]	28[a]	-	-	d6200	4	4 (4)	4 (11)	0 (0)	0 (0)
Different types of walking	30[a]	35[a]	17[a]	-	d4508[b]	4	2 (2)	1 (3)	1 (2)	0 (0)
Walking in different situations	30[a]	31[a]	25[a]	-	d4508[b]	4	2 (2)	2 (5)	0 (0)	0 (0)
Doing housework	30[a]	28[a]	-	-	d640	4	1 (1)	1 (3)	0 (0)	0 (0)
Maintaining a kneeling position	36[a]	31[a]	-	-	d4152	3	3 (3)	2 (5)	0 (0)	1 (33)

Physical function	Ranking (most often assessed =1)				ICF code	Total times assessed	Physical functions included in n (%) measures			
							Total (N=93)	SROMs (N=37)	PBOMs (N=53)	Mixed (N=3)
	ALL	Knee OA	ACL	PFPS						
Lifting and carrying, other specified	36[a]	31[a]	-	-	d4308	3	2 (2)	1 (3)	1 (2)	0 (0)
Moving around within the home	38[a]	35[a]	-	16[a]	d4600	2	2 (2)	2 (5)	0 (0)	0 (0)
Washing and drying yourself	38[a]	35[a]	-	-	d5108	2	2 (2)	2 (5)	0 (0)	0 (0)
Dance	38[a]	-	17[a]	-	d4558[b]	2	2 (2)	2 (5)	0 (0)	0 (0)
Cleaning living area	38[a]	35[a]	-	-	d6402	2	1 (1)	0 (0)	0 (0)	1 (33)
Taking care of plants, indoors and outdoors	42[a]	41[a]	-	-	d6505	1	1 (1)	0 (0)	0 (0)	1 (33)
Maintaining a lying position	42[a]	41[a]	-	-	d4150	1	1 (1)	1 (3)	0 (0)	0 (0)
Maintaining a	42[a]	41[a]	-	-	d4151	1	1 (1)	1 (3)	0 (0)	0 (0)

Physical function	Ranking (most often assessed =1)				ICF code	Total times assessed	Physical functions included in n (%) measures			
							Total (N=93)	SROMs (N=37)	PBOMs (N=53)	Mixed (N=3)
	ALL	Knee OA	ACL	PFPS						
squatting position										
Pushing with lower extremities	42[a]	41[a]	-	-	d4350	1	1 (1)	1 (3)	0 (0)	0 (0)
Kicking	42[a]	41[a]	-	-	d4351	1	1 (1)	1 (3)	0 (0)	0 (0)
Crawling	42[a]	-	25[a]	-	d4550	1	1 (1)	1 (3)	0 (0)	0 (0)
Drying oneself	42[a]	41[a]	-	-	d5102	1	1 (1)	1 (3)	0 (0)	0 (0)
Wash and dry your body	42[a]	41[a]	-	-	d5101	1	1 (1)	1 (3)	0 (0)	0 (0)
Taking off clothes	42[a]	41[a]	-	-	d5401	1	1 (1)	1 (3)	0 (0)	0 (0)
Using household appliances	42[a]	41[a]	-	-	d6403	1	1 (1)	1 (3)	0 (0)	0 (0)
Using human-powered vehicles	42[a]	-	25[a]	-	d4700	1	1 (1)	1 (3)	0 (0)	0 (0)
Participating in coitus	42[a]	41[a]	-	-	d7702	1	1 (1)	1 (3)	0 (0)	0 (0)

Physical function	Ranking (most often assessed =1)				ICF code	Total times assessed	Physical functions included in n (%) measures			
							Total (N=93)	SROMs (N=37)	PBOMs (N=53)	Mixed (N=3)
	ALL	Knee OA	ACL	PFPS						
Undertaking a simple task	42[a]	41[a]	-	-	d2100	1	1 (1)	1 (3)	0 (0)	0 (0)
Caring for hair	42[a]	41[a]	-	-	d5202	1	1 (1)	1 (3)	0 (0)	0 (0)
Caring for toenails	42[a]	41[a]	-	-	d5204	1	1 (1)	1 (3)	0 (0)	0 (0)
Stand up on tiptoes	42[a]	-	25[a]	-	d4108[b]	1	1 (1)	1 (3)	0 (0)	0 (0)
Difficulty on sitting with crossed legs	42[a]	41[a]	-	-	d4108[b]	1	1 (1)	1 (3)	0 (0)	0 (0)
Lunge forward	42[a]	-	-	=	d4108[b]	1	1 (1)	0 (0)	1 (2)	0 (0)
Reclining	42[a]	41[a]	-	-	d4108[b]	1	1 (1)	0 (0)	1 (2)	0 (0)
Knee lifts	42[a]	41[a]	-	-	d4558[b]	1	1 (1)	0 (0)	1 (2)	0 (0)
Stepping over a one-foot-high object	42[a]	41[a]	-	-	d4558[b]	1	1 (1)	1 (3)	0 (0)	0 (0)
Sidestepping short distances	42[a]	41[a]	-	-	d4558[b]	1	1 (1)	1 (3)	0 (0)	0 (0)

Physical function	Ranking (most often assessed =1)				ICF code	Total times assessed	Physical functions included in n (%) measures			
							Total (N=93)	SROMs (N=37)	PBOMs (N=53)	Mixed (N=3)
	ALL	Knee OA	ACL	PFPS						
Lateral foot scooting	42[a]	41[a]	-	-	d4558[b]	1	1 (1)	0 (0)	1 (1.9)	0 (0)
Lateral dodging	42[a]	41[a]	-	-	d4558[b]	1	1 (1)	0 (0)	1 (1.9)	0 (0)
Balance Test on a Balance Board	42[a]	41[a]	-	-	d4158[b]	1	1 (1)	0 (0)	0 (0)	1 (33)
Opening a high window above shoulder height while standing.	42[a]	41[a]	-	-	d4158[b]	1	1 (1)	1 (3)	0 (0)	0 (0)
Remunerative employment	42[a]	41[a]	-	-	d850	1	1 (1)	1 (3)	0 (0)	0 (0)
Socializing	42[a]	41[a]	-	-	d9205	1	1 (1)	1 (3)	0 (0)	0 (0)
Keep your balance when walking	42[a]	41[a]	-	-	d4158[b]	1	1 (1)	1 (3)	0 (0)	0 (0)

Physical function	Ranking (most often assessed =1)				ICF code	Total times assessed	Physical functions included in n (%) measures			
							Total (N=93)	SROMs (N=37)	PBOMs (N=53)	Mixed (N=3)
	ALL	Knee OA	ACL	PFPS						
/running on uneven ground										
Run laterally 12 meters with a crossover step twice	42[a]	-	25[a]	-	d4558[b]	1	1 (1)	0 (0.0)	1 (1.9)	0 (0)
Uncategorizable	71	-	-	-	55555	11	7 (8)	6 (17)	0	0

Abbreviations: %, percentage; ACL, anterior cruciate ligament; ICF, International Classification of Functioning, Disability and Health; N, number of measures; OA, osteoarthritis; PBOMs, performance-based outcome measures; PF, physical function; PFP, patellofemoral pain; SROMs, self-reported outcome measures

[a] denotes equal ranking.
[b] Thematic subclassification.

Supplementary Material 4. Ranking of physical functions assessed in PBOMs.

Ranking	Physical function	ICF code	Number of times assessed	n (%) of PBOMs that include the PF (N=53)
1[a]	Walking short distances	d4500	18	17 (32.1)
1[a]	Jumping	d4553	18	12 (22.6)
3	Standing	d4104	14	13 (24.5)
4	Climbing stairs	d4551	13	9 (17.0)
5	Turning, twisting, pivoting	d4108[b]	10	9 (17.0)
6[a]	Squatting	d4101	8	8 (15.1)
6[a]	Sitting	d4103	8	8 (15.1)
8	Maintaining standing	d4154	4	2 (3.8)
9	Bending	d4105	3	2 (3.8)
10[a]	Maintaining standing on one leg	d4158[b]	2	2 (3.8)
10[a]	Running	d4552	2	2 (3.8)
10[a]	Different types of walking	d4508[b]	2	1 (1.9)
13[a]	Walk long distance	d4501	1	1 (1.9)
13[a]	Lunge forward	d4108[b]	1	1 (1.9)
13[a]	Getting in/out of a vehicle	d4108[b]	1	1 (1.9)
13[a]	Reclining	d4108[b]	1	1 (1.9)
13[a]	Maintaining sitting position	d4153	1	1 (1.9)
13[a]	Lifting and carrying, other specified	d4308	1	1 (1.9)
13[a]	Carrying, moving and handling objects, other specified and unspecified	d449	1	1 (1.9)

13[a]	Knee lifts	d4558[b]	1	1 (1.9)
13[a]	Run laterally with cross-over steps	d4558[b]	1	1 (1.9)
13[a]	Lateral foot scooting	d4558[b]	1	1 (1.9)
13[a]	Lateral foot dodging	d4558[b]	1	1 (1.9)
13[a]	Rising up/sitting down	d4108[b]		1 (1.9)
Abbreviations: %, percentage; ICF, International Classification of Functioning, Disability and Health; n, number; N, number of instruments; PBOMs, performance-based outcome measures; PF, physical functions.<br><br>a: Denotes equal ranking<br>b: Thematic subclassification				

Supplementary material 5. Characteristics of the PBOMs.

Instrument (N=53)	Population	Number of physical functions assessed	Number of ICF codes mapped	Bilateral or Unilateral	Method of measurement	Measurement property assessed
5-jump test	ACL	1	1	Bilateral	Distance	Criterion validity [42]
Crossover hop	ACL	1	1	Unilateral	Distance	Criterion validity [49, 81, 104, 105], reliability [57, 115], SEM [115], responsiveness [115], MDC [115]
Single leg hop	ACL	1	1	Unilateral	Distance	Criterion validity [41, 42, 49, 81, 104, 105, 137], reliability [50, 73, 74, 103, 115], SEM [115],

Instrument (N=53)	Population	Number of physical functions assessed	Number of ICF codes mapped	Bilateral or Unilateral	Method of measurement	Measurement property assessed
						responsiveness [115], MDC [115]
Triple single hop	ACL	1	1	Unilateral	Distance	Criterion validity [49, 81, 104], reliability [115], SEM [115] responsiveness [115], MDC [115]
Y balance test	ACL	1	1	Unilateral	Distance	Criterion validity [46]
Drop jump followed by a double hop for distance	ACL	2	1	Unilateral	Distance	Reliability [50]
Vertical jump	ACL	1	1	Unilateral	Height	Reliability [23, 50]
One leg rise	ACL	1	1	Unilateral	Number of repetitions	Criterion validity [105]
Side hop	ACL	1	1	Unilateral	Number of repetitions	Criterion validity [41, 42, 105], reliability [50]
Step-Down Endurance Test	ACL	1	1	Unilateral	Number of repetitions	Criterion validity [36]
Square hop	ACL	3	1	Unilateral	Number of repetitions	Reliability [50]
6-meter time hop	ACL	1	1	Unilateral	Time	Criterion validity [49, 81, 104, 137], reliability [23, 57, 115], SEM [115], responsiveness [115], MDC [115]
Carioca test	ACL	1	1	Bilateral	Time	Reliability [73]

Instrument (N=53)	Population	Number of physical functions assessed	Number of ICF codes mapped	Bilateral or Unilateral	Method of measurement	Measurement property assessed
One leg sit to stand	ACL	1	1	Unilateral	Time	Criterion validity [141], construct validity [141]
Shuttle run test	ACL	2	2	Bilateral	Time	Reliability [73]
Stair hop	ACL	2	1	Unilateral	Time	Reliability [57]
Speedy Jump	ACL	3	1	Unilateral	Time	Content validity [53], reliability [53]
Performance-Oriented Mobility Assessment	Knee OA	6	6	Bilateral	Ability to perform	Construct validity, reliability, SEM, MDC [102]
30-second unilateral knee bending	Knee OA	1	1	Unilateral	Distance	Reliability, MDC [135]
30-Second Fast-Paced Walk Test	Knee OA	1	1	Bilateral	Distance	Construct validity, reliability, SEM, MDC [54]
2-Minute Walk Test	Knee OA	2	2	Bilateral	Distance	Reliability, SEM, MDC [26]
6-minute walk test	Knee OA	2	2	Bilateral	Distance	Content validity [78], construct validity [10, 125], reliability [10, 38], SEM [38, 98], MDC [38, 98]
Dynamic weight-bearing assessment of Pain	Knee OA	1	1	Unilateral	Number of repetitions	Construct validity [71], reliability [70], SEM [70], responsiveness[71], MDC [70], MCID [71]
2-minute step test	Knee OA	1	1	Unilateral	Number of repetitions	Construct validity, reliability, SEM [7]

Instrument (N=53)	Population	Number of physical functions assessed	Number of ICF codes mapped	Bilateral or Unilateral	Method of measurement	Measurement property assessed
Step up and down test	Knee OA	2	1	Unilateral	Number of repetitions	Construct validity, reliability, responsiveness [6]
10-meter walk test	Knee OA	1	1	Bilateral	Time	Construct validity [91], reliability [58]
20-meter walk test	Knee OA	1	1	Bilateral	Time	Criterion validity [87], reliability [95, 135], MDC [95, 135]
5-meter walking time	Knee OA	1	1	Bilateral	Time	Construct validity, internal consistency [121], responsiveness[122]
Ascending stairs	Knee OA	1	1	Bilateral	Time	Construct validity, reliability, SEM [80]
Descending stairs	Knee OA	1	1	Bilateral	Time	Construct validity, reliability, SEM [80]
8-foot walk	Knee OA	1	1	Bilateral	Time	Construct validity [80], reliability, SEM [24, 80]
Stand-to-sit time	Knee OA	1	1	Bilateral	Time	Construct validity, internal consistency [121], responsiveness[122]
Stand-to-recline time	Knee OA	1	1	Bilateral	Time	Construct validity, internal consistency [121], responsiveness[122]
40-meter fast-paced walk test	Knee OA	2	2	Bilateral	Time	Construct validity [123, 125, 126, 132],

Instrument (N=53)	Population	Number of physical functions assessed	Number of ICF codes mapped	Bilateral or Unilateral	Method of measurement	Measurement property assessed
						reliability [126, 132], SEM [126, 132], responsiveness [132], MDC [126, 132]
400-meter walk test	Knee OA	2	2	Bilateral	Time	Criterion validity [87]
Chair stands	Knee OA	2	2	Bilateral	Time	Criterion validity [87], Construct validity [80], reliability [80, 135], SEM [80], MDC [135]
Get up and go test	Knee OA	2	2	Bilateral	Time	Construct validity, reliability, SEM, MDC [110]
Timed stand test	Knee OA	2	2	Bilateral	Time	Reliability [58], responsiveness [45]
L-test	Knee OA	4	3	Bilateral	Time	Reliability, SEM, MDC [97]
Time up and go test	Knee OA	4	4	Bilateral	Time	Construct validity [78, 91, 123, 125], reliability, [5, 38], SEM [5, 38, 98], responsiveness [45], MDC [5, 38, 98]
Short Physical Performance Battery	Knee OA	5	3	Bilateral	Time	Construct validity [91]
Aggregated locomotor function	Knee OA	6	4	Bilateral	Time	Construct validity, reliability, SEM, responsiveness, MDC [88]

Instrument (N=53)	Population	Number of physical functions assessed	Number of ICF codes mapped	Bilateral or Unilateral	Method of measurement	Measurement property assessed
Physical activities restrictions	Knee OA	10	8	Bilateral	Time, Distance	Content validity, construct validity, reliability [116]
System of lower-extremity dysfunction	Knee OA	6	4	Unilateral and bilateral	Time, Number of repetitions, Ability to perform, Height	Content validity, construct validity, reliability [99]
Community balance and mobility scale	Knee OA	12	10	Unilateral and bilateral	Variable	Construct validity [127, 128], reliability [127], SEM [127]
Anteromedial lunge	PFPS	1	1	Unilateral	Number of repetitions	Reliability, SEM [82]
Balance and reach	PFPS	1	1	Unilateral	Number of repetitions	Reliability, SEM [82]
Bilateral squat	PFPS	1	1	Bilateral	Number of repetitions	Reliability, SEM [82]
6-minute step test	PFPS	1	1	Unilateral	Number of repetitions	Construct validitiy, reliability, SEM [143]
Step-down	PFPS	1	1	Unilateral	Number of repetitions	Reliability, SEM [82]
Star excursion balance test	Knee OA, ACL	1	1	Unilateral	Distance	Criterion validity [36, 41, 42], construct validity, reliability, SEM [37, 67], responsiveness [67, 93], MDC [37, 67]
30-second Chair test	Knee OA, PFPS	2	2	Bilateral	Number of repetitions	Construct validity [126, 132, 143], reliability [38, 48, 55, 126, 132, 143], SEM [38, 48, 55, 126, 132,]

Instrument (N=53)	Population	Number of physical functions assessed	Number of ICF codes mapped	Bilateral or Unilateral	Method of measurement	Measurement property assessed
						[143], responsiveness [94, 132], MDC [38, 48, 126, 132], MCID [94]
Stair-climb test	Knee OA, PFPS	1	1	Bilateral	Time	Construct validity [59, 78, 119, 123, 125, 126, 132, 143], reliability [24, 38, 55, 59, 119, 126, 132, 143], SEM [24, 38, 55, 59, 119, 126, 132, 143], responsiveness [94, 119, 132], MDC [38, 119, 126, 132] MCID [94, 119]
Abbreviations: ACL, anterior cruciate ligament; MCID, minimal clinical important difference; MDC, minimal detectable change; N, number of instruments; OA, osteoarthritis; PFPS, patellofemoral pain syndrome, SEM, standard error of measurement						

Supplementary material 6. Ranking of physical functions assessed in SROMs.

Ranking	Physical function	ICF code	Number of times assessed	n (%) of SROMs that include the PF (N=37)
1	Climbing stairs	d4551	76	31 (83.8)
2	Standing	d4104	50	25 (67.6)
3	Walking short distances	d4500	37	21 (58.3)
4[a]	Squatting	d4101	27	23 (62.2)
4[a]	Running	d4552	27	16 (44.4)
6	Sitting	d4103	26	18 (48.6)
7	Maintaining standing	d4154	21	13 (36.1)

Ranking	Physical function	ICF code	Number of times assessed	n (%) of SROMs that include the PF (N=37)
8	Jumping	d4553	19	14 (38.9)
9	Getting in/out of a vehicle	d4108[b]	18	12 (33.3)
10[a]	Kneeling	d4102	16	15 (41.6)
10[a]	Putting on footwear	d5402	16	14 (38.9)
12	Maintaining sitting	d4153	14	7 (18.9)
13	Bending	d4105	13	12 (33.3)
14	Getting in/out of the bath	d4108[b]	12	9 (24.3)
15	Walking long distances	d4501	11	5 (13.5)
16[a]	Getting in/out of a toilet	d4108[b]	10	10 (27.0)
16[a]	Taking off footwear	d5403	10	10 (27.0)
16[a]	Doing housework, heavy	d6409[b]	10	9 (24.3)
16[a]	Walking on different surfaces	d4502	10	5 (13.5)
20	Doing housework, light	d6409[b]	9	9 (24.3)
21[a]	Accelerating, decelerating while running	d4558[b]	8	8 (21.6)
21[a]	Changing directions while running	d4558[b]	8	7 (18.9)
23[a]	Lying down	d4100	7	7 (18.9)
23[a]	Changing position in bed	d4108[b]	7	4 (10.8)
25[a]	Turning, twisting, pivoting	d4108[b]	6	6 (16.7)
25[a]	Lifting	d4300	6	3 (8.1)
25[a]	Carrying in the hands	d4301	6	1 (2.7)
28	Putting on clothes	d5400	5	3 (8.1)
29[a]	Shopping	d6200	4	4 (10.8)
29[a]	Washing body parts	d5100	4	3 (8.1)
29[a]	Walking of different situations	d4508[b]	4	2 (5.4)
29[a]	Doing housework	d640	4	1 (2.7)
33	Carrying heavy objects	d449	3	3 (8.1)
34[a]	Maintaining a kneeling position	d4152	2	2 (5.4)
34[a]	Moving around within the home	d4600	2	2 (5.4)
34[a]	Washing oneself, other specified	d5108	2	2 (5.4)

Ranking	Physical function	ICF code	Number of times assessed	n (%) of SROMs that include the PF (N=37)
34[a]	Dance	d4558[b]	2	2 (5.4)
34[a]	Lifting and carrying, other specified	d4308	2	1 (2.7)
34[a]	Different types of walking	d4508[b]	2	1 (2.7)
40[a]	Stand up on tiptoes	d4108[b]	1	1 (2.7)
40[a]	Difficulty on sitting with crossed legs	d4108[b]	1	1 (2.7)
40[a]	Maintaining standing on one leg	d4158[b]	1	1 (2.7)
40[a]	Maintaining a lying position	d4150	1	1 (2.7)
40[a]	Maintaining a squatting position	d4151	1	1 (2.7)
40[a]	Pushing with lower extremities	d4350	1	1 (2.7)
40[a]	Kicking	d4351	1	1 (2.7)
40[a]	Crawling	d4550	1	1 (2.7)
40[a]	Drying oneself	d5102	1	1 (2.7)
40[a]	Taking off clothes	d5401	1	1 (2.7)
40[a]	Using household appliances	d6403	1	1 (2.7)
40[a]	Washing whole body	d5101	1	1 (2.7)
40[a]	Opening a high window above shoulder height while standing.	d4158[b]	1	1 (2.7)
40[a]	Keep your balance when walking /running on uneven ground	d4158[b]	1	1 (2.7)
40[a]	Using human-powered vehicles	d4700	1	1 (2.7)
40[a]	Stepping over a one foot high object.	d4558[b]	1	1 (2.7)
40[a]	Sidestepping short distances.	d4558[b]	1	1 (2.7)
40[a]	Undertaking a simple task	d2100	1	1 (2.7)
40[a]	Caring for hair	d5202	1	1 (2.7)
40[a]	Caring for toenails	d5204	1	1 (2.7)
40[a]	Remunerative employment	d850	1	1 (2.7)
40[a]	Socializing	d9205	1	1 (2.7)
	Unable to categorize	55555	10	5

Ranking	Physical function	ICF code	Number of times assessed	n (%) of SROMs that include the PF (N=37)
Abbreviations: %, percentage; ICF, International Classification of Functioning, Disability and Health; n, number; N, number of instruments; PF, physical functions; SROMs, self-reported outcome measures<br><br>a: Denotes equal ranking<br>b: Thematic subclassification				

Supplementary material 7. Characteristics of the SROMs.

Instrument (N=37)	Population	Number of lower limb physical functions assessed	Number of ICF codes mapped	Number and (%) of bilateral functions	Number and (%) of unilateral functions	Ranges of scores	Method of measurement	Measurement property assessed
Knee Stability in Sports/Cutting-Pivoting Ability scale	ACL	1	1	1 (100%)	0 (0%)	0-100[a]	5-point Likert scale	Construct validity, reliability, internal consistency, responsiveness, floor and ceiling effects [27]
Knee Injury and Osteoarthritis Outcome Score (KOOS) - Global	ACL	2	2	2 (100%)	0 (%)	(0-44) 0-100[a]	5-point Likert scale	Construct validity, responsiveness,

Instrument (N=37)	Population	Number of lower limb physical functions assessed	Number of ICF codes mapped	Number and (%) of bilateral functions	Number and (%) of unilateral functions	Ranges of scores	Method of measurement	Measurement property assessed
								floor and ceiling effect [64]
The International Knee Documentation Committee (IKDC) Subjective Knee Form – 8	ACL	5	5	4 (80%)	1 (20%)	0-100[a]	5-point Likert scale	Construct validity, reliability [40]
Knee Numeric-Entity Evaluation Score	ACL	14	13	14 (100%)	0 (0%)	0–3 for each item.<br>Subscale scored Separately[b]	4-point Likert scale	Content validity [30], construct validity [31], responsiveness [32]
Patient-Reported Outcomes Measurement Information System (PROMIS) Mobility	ACL	43	17	42 (98%)	1 (2%)	0-100%[a]	5-point Likert scale	Construct validity, floor and ceiling effect [35]
KOOS-Child	ACL (paediatric)	23	21	22 (95%)	1 (5%)	0%-100%[a]	5-point Likert scale	Content validity [101]
Difficulties in daily life of patients with knee osteoarthritis scale	Knee OA	4	4	4 (100%)	0 (0%)	0-100[b]	5-point Likert scale	Content validity, construct validity, internal consistency [129]

Instrument (N=37)	Population	Number of lower limb physical functions assessed	Number of ICF codes mapped	Number and (%) of bilateral functions	Number and (%) of unilateral functions	Ranges of scores	Method of measurement	Measurement property assessed
Lequesne index	Knee OA	4	3	4 (100%)	0 (0%)	0-24[b]	Variable Likert scale	Content validity [79], construct validity [43, 44], reliability [43], internal consistency [44], responsiveness [45, 47, 84]
Oxford Knee Score	Knee OA	5	5	5 (100%)	0 (0%)	0-48[a]	5-point Likert scale	Construct validity [33, 51, 75], reliability, internal consistency, SEM, responsiveness, MCD, MCID [75]
Aberdeen Activity Limitation scale modified	Knee OA	7	6	7 (100%)	0 (0%)	5-35[b]	5-point Likert scale	Construct validity [113]
KOOS-Knee flexion domain	Knee OA	7	6	7 (100%)	0 (0%)	0%-100%[b]	5-point Likert scale	Content validity, construct validity [83]
KOOS-PS	Knee OA	7	6	6 (86%)	1 (14%)	0-100[a,b]	5-point Likert scale	Construct validity [106], reliability [106],

Instrument (N=37)	Population	Number of lower limb physical functions assessed	Number of ICF codes mapped	Number and (%) of bilateral functions	Number and (%) of unilateral functions	Ranges of scores	Method of measurement	Measurement property assessed
								[120], internal consistency [106], responsiveness [84], MCID [120]
Shortened version of the Western Ontario and McMaster Universities Arthritis (WOMAC)	Knee OA	7	6	7 (100%)	0 (0%)	0-48[b]<br>0-120[b]<br>0-100%[b]	5-point Likert scale<br>11-point numeric rating scale	Criterion validity [138], construct validity [142], reliability [138], internal consistency [142], SEM [1], responsiveness [142], MDC, MCID [1]
PROMIS Short Form Physical Function	Knee OA	8	5	8 (100%)	0 (0%)	0-100%[a]	5-point Likert scale	Construct validity, floor and ceiling effect [39]
Japanese Knee Osteoarthritis Measure	Knee OA	10	8	10 (100%)	0 (0%)	0-92[b]	Visual analogue scale and Variable Likert scale	Content validity, construct validity, reliability [2]
Walking Impairment Questionnaire	Knee OA	13	3	13 (100%)	0 (0%)	0-100[a]	5-point Likert scale	Construct validity, reliability,

Instrument (N=37)	Population	Number of lower limb physical functions assessed	Number of ICF codes mapped	Number and (%) of bilateral functions	Number and (%) of unilateral functions	Ranges of scores	Method of measurement	Measurement property assessed
								internal consistency [29]
Aberdeen Activity Limitation scale	Knee OA	15	13	15 (100%)	0 (0%)	15-75[b]	5-point Likert scale	Content validity, construct validity, reliability [112]
Japanese KOOS	Knee OA	15	12	15 (100%)	0 (0%)	0%-100%[b]	5-point Likert scale	Content validity, construct validity [83]
Animated Activity Questionnaire	Knee OA	17	8	17 (100%)	0 (0%)	0-100%[b]	Variable Likert scale	Content validity [131], construct validity [107, 108, 131], reliability, internal consistency, SEM, MDC [108], responsiveness, MCID [109]
WOMAC	Knee OA	17	15	17 (100%)	0 (0%)	0-96[b]<br>0-240[b]<br>0-100%[a]	5-point Likert scale<br>11-point numeric rating scale	Content validity [13], construct validity [13, 14, 21], reliability [14, 24, 140], internal consistency [21], SEM [24, 140],

Instrument (N=37)	Population	Number of lower limb physical functions assessed	Number of ICF codes mapped	Number and (%) of bilateral functions	Number and (%) of unilateral functions	Ranges of scores	Method of measurement	Measurement property assessed
								responsiveness [9, 14, 21, 45, 47, 140], MDC [140], MCID [133, 140]
Arabic Knee Osteoarthritis Outcome Measure	Knee OA	19	15	19 (100%)	0 (0%)	0-100[a]	5-point Likert scale	Content validity, construct validity, reliability, internal consistency [8]
Korean knee score	Knee OA	19	16	19 (100%)	0 (0%)	0-164[a]	5-point Likert scale	Content validity, construct validity, reliability, responsiveness, floor and ceiling effect [69]
KOOS Fun-Sport	Knee OA	21	19	21 (100%)	0 (0%)	0%-100%[a]	5-point Likert scale	Construct validity [124]
Osteoarthritis Function Computerized Adaptive Test	Knee OA	124	40	120 (97%)	4 (3%)	0-100[a]	5-point Likert scale	Content validity, construct validity, reliability, floor and ceiling

Instrument (N=37)	Population	Number of lower limb physical functions assessed	Number of ICF codes mapped	Number and (%) of bilateral functions	Number and (%) of unilateral functions	Ranges of scores	Method of measurement	Measurement property assessed
								effect [66], responsiveness [25]
Anterior Knee Pain Scale 6-item	PFPS	3	3	3 (100%)	0 (0%)	0-6[a]	Dichotomous scale	Construct validity [96]
Anterior Knee Pain Scale	PFPS	6	6	6 (100%)	0 (0%)	0-100[a]	Variable Likert scale	Content validity [76], construct validity [34, 76], SEM [34, 136], responsiveness [34, 136], MDC [136], MCID [34]
Functional index questionnaire	PFPS	8	7	8 (100%)	0 (0%)	0-16[a]	3-point Likert scale	Construct validity [34], reliability [17, 28, 34], SEM [17], responsiveness, MCID [34]
Samsung Medical Center patellofemoral scoring system	PFPS	9	5	9 (100%)	0 (0%)	0-170[b]	5-point Likert scale	Content validity, construct validity, reliability, internal consistency, floor and ceiling effect [77]

Instrument (N=37)	Population	Number of lower limb physical functions assessed	Number of ICF codes mapped	Number and (%) of bilateral functions	Number and (%) of unilateral functions	Ranges of scores	Method of measurement	Measurement property assessed
Munich Knee Questionnaire	Knee OA, ACL	10	8	10 (100%)	0 (0%)	0-100%[a]	11-point Likert scale	Content validity, construct validity, reliability, internal consistency, responsiveness, floor and ceiling effect [12]
Lower Limb Tasks Questionnaire	Knee OA, ACL	20	13	20 (100%)	0 (0%)	0-80[a]	5-point Likert scale	Content validity [90], construct validity [89, 90], reliability, internal consistency, SEM [90], responsiveness [89, 90], floor and ceiling effect, MCID [90]
Lower extremity functional scale	Knee OA, PFPS	20	17	19 (95%)	1 (5%)	0-80[a]	5-point Likert scale	Content validity [18], construct validity [18, 123], reliability, SEM, [18, 136, 140], internal

Instrument (N=37)	Population	Number of lower limb physical functions assessed	Number of ICF codes mapped	Number and (%) of bilateral functions	Number and (%) of unilateral functions	Ranges of scores	Method of measurement	Measurement property assessed
								consistency [18], responsiveness [84, 136, 140], MCD [18, 136, 140], MCID [18, 140]
Lysholm Knee Scoring Scale	ACL, PFPS	2	2	2 (100%)	0 (0%)	0-100[a]	Variable Likert scale	Content validity [130], construct validity [22, 86, 114, 130], reliability [16, 22, 86], internal consistency [22], SEM [22], responsiveness [32, 86], floor and ceiling effect [114], MDC [22], substantial clinical benefit [65]
Hughston Clinic questionnaire	ACL, PFPS	16	15	15 (94%)	1 (6%)	0-100%[a]	10-cm Visual Analogue Scale	Construct validity [56], reliability [17], SEM [17]
Pedi-IKDC	ACL, PFPS (paediatric)	9	8	7 (78%)	2 (22%)	0-100%[a]	5-point Likert scale	Construct validity, reliability, internal

Instrument (N=37)	Population	Number of lower limb physical functions assessed	Number of ICF codes mapped	Number and (%) of bilateral functions	Number and (%) of unilateral functions	Ranges of scores	Method of measurement	Measurement property assessed
								consistency, responsiveness, floor and ceiling effect [72]
Knee Outcome Survey Activities of Daily Living Scale	Knee OA, ACL, PFPS	8	7	8 (100%)	0 (0%)	0=100%[a]	6-point Likert scale	Content validity [63], criterion validity [137], construct validity [63, 86], reliability [63, 86, 140], interntal consistency [63], SEM [140], responsiveness [63, 86, 111, 140], MDC [140], MCID [111, 140]
IKDC	Knee OA, ACL, PFPS	9	8	8 (87%)	1 (13%)	18-87[a]<br>0-100%[a]	5-point Likert scale	Content validity [61, 134], criterion validity [36, 42, 104], construct validity [52, 61, 114, 118, 134, 139], reliability [61, 134], internal consistency [52, 118], SEM [134],

Instrument (N=37)	Population	Number of lower limb physical functions assessed	Number of ICF codes mapped	Number and (%) of bilateral functions	Number and (%) of unilateral functions	Ranges of scores	Method of measurement	Measurement property assessed
								responsiveness [32, 62, 134], substantial clinical benefit [65], floor and ceiling effect [114, 134], MDC [134]
KOOS	Knee OA, ACL, PFPS	21	19	21 (100%)	0 (0%)	0%-100%[a]	5-point Likert scale	Content validity [117, 134], criterion validity [42], construct validity [85, 117, 134], reliability [117, 134], SEM [98, 134], responsiveness [32, 117, 134], MDC [98, 134], floor and ceiling effect [134], MCID [60, 92]

Abbreviations: ACL, anterior cruciate ligament; MCID, minimal clinical important difference; MDC, minimal detectable change; N, number of instruments; OA, osteoarthritis; PFPS, patellofemoral pain syndrome; SEM, standard error of measurement

a: Higher scores mean higher functional ability
b: Higher scores mean lower functional ability

References

1. Abbott JH, Hobbs C, Gwynne-Jones D. The ShortMAC: Minimum Important Change of a Reduced Version of the Western Ontario and McMaster Universities Osteoarthritis Index. *J Orthop Sports Phys Ther*. 2018;48:81-86.
2. Akai M, Doi T, Fujino K, Iwaya T, Kurosawa H, Nasu T. An outcome measure for Japanese people with knee osteoarthritis. *J Rheumatol*. 2005;32:1524-1532.
3. Akinpelu AO, Odole AC, Adegoke BOA, Adeyini AF. Development and initial validation of the ibadan knee/hip osteoarthritis outcome measure. *2007*. 2007;63:6.
4. Akinpelu AO, Odole AC, Raheem S. Minimal Clinically Important Difference of Ibadan Knee Hip OsteoArthritis Outcome Measure (IKHOAM)- A Cross-Sectional Study. 2011.
5. Alghadir A, Anwer S, Brismée JM. The reliability and minimal detectable change of Timed Up and Go test in individuals with grade 1-3 knee osteoarthritis. *BMC Musculoskelet Disord*. 2015;16:174.
6. Almeida GPL, Monteiro IO, Dantas RGO, Tavares MLA, Lima POP. Reliability, validity and responsiveness of the Step Up and Down (StUD) test for individuals with symptomatic knee osteoarthritis. *Musculoskelet Sci Pract*. 2021;56:102454.
7. Almeida TF, Dibai-Filho AV, de Freitas Thomaz F, Lima EAA, Cabido CET. Construct validity and reliability of the 2-minute step test in patients with knee osteoarthritis. *BMC Musculoskeletal Disorders*. 2022;23:159.
8. Alotaibi AD, Vennu V, BinNasser AS, et al. Development and Validation of a New Patient-Reported Outcome Measure in the Arabic Language for Patients with Knee Osteoarthritis in Saudi Arabia. *Patient Prefer Adherence*. 2023;17:187-198.
9. Angst F, Aeschlimann A, Steiner W, Stucki G. Responsiveness of the WOMAC osteoarthritis index as compared with the SF-36 in patients with osteoarthritis of the legs undergoing a comprehensive rehabilitation intervention. *Ann Rheum Dis*. 2001;60:834-840.
10. Ateef M, Kulandaivelan S, Tahseen S. Test–retest Reliability and Correlates of 6-minute Walk Test in Patients with Primary Osteoarthritis of Knees. *Indian Journal of Rheumatology*. 2016;11:192-196.
11. Barber-Westin SD, Noyes FR, McCloskey JW. Rigorous statistical reliability, validity, and responsiveness testing of the Cincinnati knee rating system in 350 subjects with uninjured, injured, or anterior cruciate ligament-reconstructed knees. *Am J Sports Med*. 1999;27:402-416.
12. Beirer M, Fiedler N, Huber S, et al. The Munich Knee Questionnaire: Development and Validation of a New Patient-Reported Outcome Measurement Tool for Knee Disorders. *Arthroscopy*. 2015;31:1522-1529.
13. Bellamy N, Buchanan WW. A preliminary evaluation of the dimensionality and clinical importance of pain and disability in osteoarthritis of the hip and knee. *Clin Rheumatol*. 1986;5:231-241.
14. Bellamy N, Buchanan WW, Goldsmith CH, Campbell J, Stitt LW. Validation study of WOMAC: a health status instrument for measuring clinically important patient relevant outcomes to antirheumatic drug therapy in patients with osteoarthritis of the hip or knee. *J Rheumatol*. 1988;15:1833-1840.
15. Bellamy N, Wilson C, Hendrikz J, et al. Osteoarthritis Index delivered by mobile phone (m-WOMAC) is valid, reliable, and responsive. *J Clin Epidemiol*. 2011;64:182-190.

16. Bengtsson J, Möllborg J, Werner S. A study for testing the sensitivity and reliability of the Lysholm knee scoring scale. *Knee Surg Sports Traumatol Arthrosc*. 1996;4:27-31.
17. Bennell K, Bartam S, Crossley K, Green S. Outcome measures in patellofemoral pain syndrome: test retest reliability and inter-relationships. *Physical Therapy in Sport*. 2000;1:32-41.
18. Binkley JM, Stratford PW, Lott SA, Riddle DL. The Lower Extremity Functional Scale (LEFS): scale development, measurement properties, and clinical application. North American Orthopaedic Rehabilitation Research Network. *Phys Ther*. 1999;79:371-383.
19. Björklund K, Andersson L, Dalén N. Validity and responsiveness of the test of athletes with knee injuries: the new criterion based functional performance test instrument. *Knee Surg Sports Traumatol Arthrosc*. 2009;17:435-445.
20. Björklund K, Sköld C, Andersson L, Dalén N. Reliability of a criterion-based test of athletes with knee injuries; where the physiotherapist and the patient independently and simultaneously assess the patient's performance. *Knee Surg Sports Traumatol Arthrosc*. 2006;14:165-175.
21. Brazier JE, Harper R, Munro J, Walters SJ, Snaith ML. Generic and condition-specific outcome measures for people with osteoarthritis of the knee. *Rheumatology (Oxford)*. 1999;38:870-877.
22. Briggs KK, Lysholm J, Tegner Y, Rodkey WG, Kocher MS, Steadman JR. The reliability, validity, and responsiveness of the Lysholm score and Tegner activity scale for anterior cruciate ligament injuries of the knee: 25 years later. *Am J Sports Med*. 2009;37:890-897.
23. Brosky JA, Jr., Nitz AJ, Malone TR, Caborn DN, Rayens MK. Intrarater reliability of selected clinical outcome measures following anterior cruciate ligament reconstruction. *J Orthop Sports Phys Ther*. 1999;29:39-48.
24. C. Davey R, Edwards SM, Cochrane T. Test–retest Reliability of Lower Extremity Functional and Self-reported Measures in Elderly with Osteoarthritis. *Advances in Physiotherapy*. 2003;5:155-160.
25. Chang F-H, Jette AM, Slavin MD, Baker K, Ni P, Keysor JJ. Detecting functional change in response to exercise in knee osteoarthritis: a comparison of two computerized adaptive tests. *BMC Musculoskeletal Disorders*. 2018;19:29.
26. Chaudhary S, Suhail A. Test-Retest Reliability and Minimum Detectable Change of 2-Minute Walk Test among Individuals with Knee Osteoarthritis. *Journal of Clinical & Diagnostic Research*. 2021;15:4-7.
27. Chen J, Cho E, Xu C, Zhao J. A New Rating Scale for the Rapid Evaluation of High-Level Sports Ability. *Orthop J Sports Med*. 2020;8:2325967120964883.
28. Chesworth BM, Culham E, Tata GE, Peat M. Validation of outcome measures in patients with patellofemoral syndrome. *J Orthop Sports Phys Ther*. 1989;10:302-308.
29. Collins E, O'Connell S, Jelinek C, Miskevics S, Budiman-Mak E. Evaluation of psychometric properties of Walking Impairment Questionnaire in overweight patients with osteoarthritis of knee. *J Rehabil Res Dev*. 2008;45:559-566.
30. Comins JD, Krogsgaard MR, Brodersen J. Development of the Knee Numeric-Entity Evaluation Score (KNEES-ACL): a condition-specific questionnaire. *Scand J Med Sci Sports*. 2013;23:e293-301.
31. Comins JD, Krogsgaard MR, Kreiner S, Brodersen J. Dimensionality of the Knee Numeric-Entity Evaluation Score (KNEES-ACL): a condition-specific questionnaire. *Scand J Med Sci Sports*. 2013;23:e302-312.
32. Comins JD, Siersma VD, Lind M, Jakobsen BW, Krogsgaard MR. KNEES-ACL has superior responsiveness compared to the most commonly used patient-reported outcome measures for anterior cruciate ligament injury. *Knee Surg Sports Traumatol Arthrosc*. 2018;26:2438-2446.

33. Conaghan PG, Emerton M, Tennant A. Internal construct validity of the Oxford Knee Scale: evidence from Rasch measurement. *Arthritis Rheum*. 2007;57:1363-1367.
34. Crossley KM, Bennell KL, Cowan SM, Green S. Analysis of outcome measures for persons with patellofemoral pain: which are reliable and valid? *Arch Phys Med Rehabil*. 2004;85:815-822.
35. Day MA, Hancock KJ, Antao VC, et al. Preoperative Evaluation of the Lower Extremity-Specific PROMIS Mobility Bank in Patients with ACL Tears. *Arthrosc Sports Med Rehabil*. 2021;3:e1025-e1029.
36. de Fontenay B, Van Cant J, Gokeler A, Roy JS. Reintroduction of Running After Anterior Cruciate Ligament Reconstruction With a Hamstrings Graft: Can We Predict Short-Term Success? *J Athl Train*. 2022;57:540-546.
37. Dobija L, Reynaud V, Pereira B, et al. Measurement properties of the Star Excursion Balance Test in patients with ACL deficiency. *Phys Ther Sport*. 2019;36:7-13.
38. Dobson F, Hinman RS, Hall M, et al. Reliability and measurement error of the Osteoarthritis Research Society International (OARSI) recommended performance-based tests of physical function in people with hip and knee osteoarthritis. *Osteoarthritis Cartilage*. 2017;25:1792-1796.
39. Driban JB, Morgan N, Price LL, Cook KF, Wang C. Patient-Reported Outcomes Measurement Information System (PROMIS) instruments among individuals with symptomatic knee osteoarthritis: a cross-sectional study of floor/ceiling effects and construct validity. *BMC Musculoskelet Disord*. 2015;16:253.
40. Duckett T, Fox CM, Hart JM, Norte GE. Rationale for a Parsimonious Measure of Subjective Knee Function Among Individuals With Anterior Cruciate Ligament Reconstruction: A Rasch Analysis. *J Athl Train*. 2021;56:1340-1348.
41. Fältström A, Hägglund M, Hedevik H, Kvist J. Poor Validity of Functional Performance Tests to Predict Knee Injury in Female Soccer Players With or Without Anterior Cruciate Ligament Reconstruction. *Am J Sports Med*. 2021;49:1441-1450.
42. Fältström A, Kvist J, Bittencourt NFN, Mendonça LD, Hägglund M. Clinical Risk Profile for a Second Anterior Cruciate Ligament Injury in Female Soccer Players After Anterior Cruciate Ligament Reconstruction. *Am J Sports Med*. 2021;49:1421-1430.
43. Faucher M, Poiraudeau S, Lefevre-Colau MM, Rannou F, Fermanian J, Revel M. Assessment of the test-retest reliability and construct validity of a modified Lequesne index in knee osteoarthritis. *Joint Bone Spine*. 2003;70:521-525.
44. Franchignoni F, Salaffi F, Giordano A, Ciapetti A, Carotti M, Ottonello M. Psychometric properties of self-administered Lequesne Algofunctional Indexes in patients with hip and knee osteoarthritis: an evaluation using classical test theory and Rasch analysis. *Clin Rheumatol*. 2012;31:113-121.
45. French HP, Fitzpatrick M, FitzGerald O. Responsiveness of physical function outcomes following physiotherapy intervention for osteoarthritis of the knee: an outcome comparison study. *Physiotherapy*. 2011;97:302-308.
46. Garrison JC, Bothwell JM, Wolf G, Aryal S, Thigpen CA. Y BALANCE TEST™ ANTERIOR REACH SYMMETRY AT THREE MONTHS IS RELATED TO SINGLE LEG FUNCTIONAL PERFORMANCE AT TIME OF RETURN TO SPORTS FOLLOWING ANTERIOR CRUCIATE LIGAMENT RECONSTRUCTION. *Int J Sports Phys Ther*. 2015;10:602-611.
47. Gentelle-Bonnassies S, Le Claire P, Mezieres M, Ayral X, Dougados M. Comparison of the responsiveness of symptomatic outcome measures in knee osteoarthritis. *Arthritis Care Res*. 2000;13:280-285.

48. Gill S, Hely R, Page RS, Hely A, Harrison B, Landers S. Thirty second chair stand test: Test-retest reliability, agreement and minimum detectable change in people with early-stage knee osteoarthritis. *Physiother Res Int*. 2022;27:e1957.
49. Grindem H, Logerstedt D, Eitzen I, et al. Single-legged hop tests as predictors of self-reported knee function in nonoperatively treated individuals with anterior cruciate ligament injury. *Am J Sports Med*. 2011;39:2347-2354.
50. Gustavsson A, Neeter C, Thomeé P, et al. A test battery for evaluating hop performance in patients with an ACL injury and patients who have undergone ACL reconstruction. *Knee Surg Sports Traumatol Arthrosc*. 2006;14:778-788.
51. Harris K, Dawson J, Doll H, et al. Can pain and function be distinguished in the Oxford Knee Score in a meaningful way? An exploratory and confirmatory factor analysis. *Qual Life Res*. 2013;22:2561-2568.
52. Higgins LD, Taylor MK, Park D, et al. Reliability and validity of the International Knee Documentation Committee (IKDC) Subjective Knee Form. *Joint Bone Spine*. 2007;74:594-599.
53. Hildebrandt C, Müller L, Zisch B, Huber R, Fink C, Raschner C. Functional assessments for decision-making regarding return to sports following ACL reconstruction. Part I: development of a new test battery. *Knee Surg Sports Traumatol Arthrosc*. 2015;23:1273-1281.
54. Hoglund LT, Folkins E, Pontiggia L, Knapp MW. The Validity, Reliability, Measurement Error, and Minimum Detectable Change of the 30-Second Fast-Paced Walk Test in Persons with Knee Osteoarthritis: A Novel Test of Short-Distance Walking Ability. *ACR Open Rheumatol*. 2019;1:279-286.
55. Holm PM, Nyberg M, Wernbom M, Schrøder HM, Skou ST. Intrarater Reliability and Agreement of Recommended Performance-Based Tests and Common Muscle Function Tests in Knee Osteoarthritis. *J Geriatr Phys Ther*. 2021;44:144-152.
56. Hooper DM, Morrissey MC, Drechsler WI, McDermott M, McAuliffe TB. Validation of the Hughston Clinic subjective knee questionnaire using gait analysis. *Med Sci Sports Exerc*. 2001;33:1456-1462.
57. Hopper DM, Goh SC, Wentworth LA, et al. Test–retest reliability of knee rating scales and functional hop tests one year following anterior cruciate ligament reconstruction. *Physical Therapy in Sport*. 2002;3:10-18.
58. Howe T, Oldham J. Functional tests in elderly osteoarthritic subjects: variability of performance. *Nurs Stand*. 1995;9:35-38.
59. Iijima H, Shimoura K, Eguchi R, Aoyama T, Takahashi M. Concurrent validity and measurement error of stair climb test in people with pre-radiographic to mild knee osteoarthritis. *Gait Posture*. 2019;68:335-339.
60. Ingelsrud LH, Terwee CB, Terluin B, et al. Meaningful Change Scores in the Knee Injury and Osteoarthritis Outcome Score in Patients Undergoing Anterior Cruciate Ligament Reconstruction. *Am J Sports Med*. 2018;46:1120-1128.
61. Irrgang JJ, Anderson AF, Boland AL, et al. Development and validation of the international knee documentation committee subjective knee form. *Am J Sports Med*. 2001;29:600-613.
62. Irrgang JJ, Anderson AF, Boland AL, et al. Responsiveness of the International Knee Documentation Committee Subjective Knee Form. *Am J Sports Med*. 2006;34:1567-1573.
63. Irrgang JJ, Snyder-Mackler L, Wainner RS, Fu FH, Harner CD. Development of a patient-reported measure of function of the knee. *J Bone Joint Surg Am*. 1998;80:1132-1145.
64. Jacobs CA, Peabody MR, Lattermann C, et al. Development of the KOOS(global) Platform to Measure Patient-Reported Outcomes After Anterior Cruciate Ligament Reconstruction. *Am J Sports Med*. 2018;46:2915-2921.

65. Jeon YS, Lee JW, Kim SH, Kim SG, Kim YH, Bae JH. Determining the Substantial Clinical Benefit Values for Patient-Reported Outcome Scores After Primary ACL Reconstruction. *Orthop J Sports Med*. 2022;10:23259671221091795.
66. Jette AM, McDonough CM, Ni P, et al. A functional difficulty and functional pain instrument for hip and knee osteoarthritis. *Arthritis Res Ther*. 2009;11:R107.
67. Kanko LE, Birmingham TB, Bryant DM, et al. The star excursion balance test is a reliable and valid outcome measure for patients with knee osteoarthritis. *Osteoarthritis Cartilage*. 2019;27:580-585.
68. Kersten P, White PJ, Tennant A. The visual analogue WOMAC 3.0 scale--internal validity and responsiveness of the VAS version. *BMC Musculoskelet Disord*. 2010;11:80.
69. Kim JG, Ha JK, Han SB, Kim TK, Lee MC. Development and validation of a new evaluation system for patients with a floor-based lifestyle: the Korean knee score. *Clin Orthop Relat Res*. 2013;471:1539-1547.
70. Klokker L, Christensen R, Osborne R, et al. Dynamic weight-bearing assessment of pain in knee osteoarthritis: a reliability and agreement study. *Qual Life Res*. 2015;24:2985-2992.
71. Klokker L, Christensen R, Wæhrens EE, et al. Dynamic weight-bearing assessment of pain in knee osteoarthritis: construct validity, responsiveness, and interpretability in a research setting. *Health and Quality of Life Outcomes*. 2016;14:91.
72. Kocher MS, Smith JT, Iversen MD, et al. Reliability, validity, and responsiveness of a modified International Knee Documentation Committee Subjective Knee Form (Pedi-IKDC) in children with knee disorders. *Am J Sports Med*. 2011;39:933-939.
73. Kong DH, Yang SJ, Ha JK, Jang SH, Seo JG, Kim JG. Validation of functional performance tests after anterior cruciate ligament reconstruction. *Knee Surg Relat Res*. 2012;24:40-45.
74. Kramer JF, Nusca D, Fowler P, Webster-Bogaert S. Test-Retest Reliability of the One-Leg Hop Test Following ACL Reconstruction. *Clinical Journal of Sport Medicine*. 1992;2:
75. Kristina KH, Jill D, Luke DJ, David JB, Andrew JP. Extending the use of PROMs in the NHS—using the Oxford Knee Score in patients undergoing non-operative management for knee osteoarthritis: a validation study. *BMJ Open*. 2013;3:e003365.
76. Kujala UM, Jaakkola LH, Koskinen SK, Taimela S, Hurme M, Nelimarkka O. Scoring of patellofemoral disorders. *Arthroscopy: The Journal of Arthroscopic & Related Surgery*. 1993;9:159-163.
77. Lee CH, Ha CW, Kim S, Kim M, Song YJ. A novel patellofemoral scoring system for patellofemoral joint status. *J Bone Joint Surg Am*. 2013;95:620-626.
78. Lee SH, Kao CC, Liang HW, Wu HT. Validity of the Osteoarthritis Research Society International (OARSI) recommended performance-based tests of physical function in individuals with symptomatic Kellgren and Lawrence grade 0-2 knee osteoarthritis. *BMC Musculoskelet Disord*. 2022;23:1040.
79. Lequesne MG, Mery C, Samson M, Gerard P. Indexes of severity for osteoarthritis of the hip and knee. Validation--value in comparison with other assessment tests. *Scand J Rheumatol Suppl*. 1987;65:85-89.
80. Lin YC, Davey RC, Cochrane T. Tests for physical function of the elderly with knee and hip osteoarthritis. *Scand J Med Sci Sports*. 2001;11:280-286.
81. Logerstedt D, Grindem H, Lynch A, et al. Single-legged hop tests as predictors of self-reported knee function after anterior cruciate ligament reconstruction: the Delaware-Oslo ACL cohort study. *Am J Sports Med*. 2012;40:2348-2356.

82. Loudon JK, Wiesner D, Goist-Foley HL, Asjes C, Loudon KL. Intrarater Reliability of Functional Performance Tests for Subjects With Patellofemoral Pain Syndrome. *J Athl Train*. 2002;37:256-261.
83. Lyman S, Omori G, Nakamura N, et al. Development and validation of a culturally relevant Japanese KOOS. *J Orthop Sci*. 2019;24:514-520.
84. Mahler E, Cuperus N, Bijlsma J, et al. Responsiveness of four patient-reported outcome measures to assess physical function in patients with knee osteoarthritis. *Scand J Rheumatol*. 2016;45:518-527.
85. Marmura H, Tremblay PF, Getgood AMJ, Bryant DM. The Knee Injury and Osteoarthritis Outcome Score Does Not Have Adequate Structural Validity for Use With Young, Active Patients With ACL Tears. *Clin Orthop Relat Res*. 2022;480:1342-1350.
86. Marx RG, Jones EC, Allen AA, et al. Reliability, validity, and responsiveness of four knee outcome scales for athletic patients. *J Bone Joint Surg Am*. 2001;83:1459-1469.
87. Master H, Thoma LM, Christiansen MB, Polakowski E, Schmitt LA, White DK. Minimum Performance on Clinical Tests of Physical Function to Predict Walking 6,000 Steps/Day in Knee Osteoarthritis: An Observational Study. *Arthritis Care Res (Hoboken)*. 2018;70:1005-1011.
88. McCarthy CJ, Oldham JA. The reliability, validity and responsiveness of an aggregated locomotor function (ALF) score in patients with osteoarthritis of the knee. *Rheumatology (Oxford)*. 2004;43:514-517.
89. McKay C, Prapavessis H, McNair P. Comparing the lower limb tasks questionnaire to the Western Ontario and McMaster Universities Osteoarthritis Index: agreement, responsiveness, and convergence with physical performance for knee osteoarthritis patients. *Arch Phys Med Rehabil*. 2013;94:474-479.
90. McNair PJ, Prapavessis H, Collier J, Bassett S, Bryant A, Larmer P. The lower-limb tasks questionnaire: an assessment of validity, reliability, responsiveness, and minimal important differences. *Arch Phys Med Rehabil*. 2007;88:993-1001.
91. Mehta SP, Morelli N, Prevatte C, White D, Oliashirazi A. Validation of Physical Performance Tests in Individuals with Advanced Knee Osteoarthritis. *Hss j*. 2019;15:261-268.
92. Mills KA, Naylor JM, Eyles JP, Roos EM, Hunter DJ. Examining the Minimal Important Difference of Patient-reported Outcome Measures for Individuals with Knee Osteoarthritis: A Model Using the Knee Injury and Osteoarthritis Outcome Score. *J Rheumatol*. 2016;43:395-404.
93. Mostafaee N, Negahban H, Shaterzadeh Yazdi MJ, Goharpey S, Mehravar M, Pirayeh N. Responsiveness of Impairment-based Outcome Measures in Individuals With Anterior Cruciate Ligament Reconstruction Following Physiotherapy. *USWR*. 2021;22:228-245.
94. Mostafaee N, Rashidi F, Negahban H, Ebrahimzadeh MH. Responsiveness and minimal important changes of the OARSI core set of performance-based measures in patients with knee osteoarthritis following physiotherapy intervention. *Physiother Theory Pract*. 2022;1-12.
95. Motyl JM, Driban JB, McAdams E, Price LL, McAlindon TE. Test-retest reliability and sensitivity of the 20-meter walk test among patients with knee osteoarthritis. *BMC Musculoskelet Disord*. 2013;14:166.
96. Myer GD, Barber Foss KD, Gupta R, Hewett TE, Ittenbach RF. Analysis of patient-reported anterior knee pain scale: implications for scale development in children and adolescents. *Knee Surg Sports Traumatol Arthrosc*. 2016;24:653-660.
97. Nalbant A, Unver B, Karatosun V. Test-retest reliability of the L-Test in patients with advanced knee osteoarthritis. *Physiother Theory Pract*. 2022;38:2983-2987.

98. Naylor JM, Hayen A, Davidson E, et al. Minimal detectable change for mobility and patient-reported tools in people with osteoarthritis awaiting arthroplasty. *BMC Musculoskelet Disord*. 2014;15:235.
99. Oberg U, Oberg B, Oberg T. Validity and reliability of a new assessment of lower-extremity dysfunction. *Phys Ther*. 1994;74:861-871.
100. Odole AC, Odunaiya NA, Akinpelu AO. Ibadan knee/hip osteoarthritis outcome measure: process of development. *Ann Ib Postgrad Med*. 2013;11:71-76.
101. Örtqvist M, Roos EM, Broström EW, Janarv PM, Iversen MD. Development of the Knee Injury and Osteoarthritis Outcome Score for children (KOOS-Child): comprehensibility and content validity. *Acta Orthop*. 2012;83:666-673.
102. Parveen H, Noohu MM. Evaluation of psychometric properties of Tinetti performance-oriented mobility assessment scale in subjects with knee osteoarthritis. *Hong Kong Physiother J*. 2017;36:25-32.
103. Paterno MV, Greenberger HB. The test-retest reliability of a one legged hop for distance in young adults with and without ACL reconstruction. *Isokinetics and Exercise Science*. 1996;6:1-6.
104. Paterno MV, Rauh MJ, Thomas S, Hewett TE, Schmitt LC. Return-to-Sport Criteria After Anterior Cruciate Ligament Reconstruction Fail to Identify the Risk of Second Anterior Cruciate Ligament Injury. *J Athl Train*. 2022;57:937-945.
105. Patterson B, Culvenor AG, Barton CJ, et al. Poor functional performance 1 year after ACL reconstruction increases the risk of early osteoarthritis progression. *Br J Sports Med*. 2020;54:546-553.
106. Perruccio AV, Stefan Lohmander L, Canizares M, et al. The development of a short measure of physical function for knee OA KOOS-Physical Function Shortform (KOOS-PS) - an OARSI/OMERACT initiative. *Osteoarthritis Cartilage*. 2008;16:542-550.
107. Peter WF, Dagfinrud HS, Østerås N, Terwee CB. Animated Activity Questionnaire (AAQ), a new method of self-reporting activity limitations in patients with hip and knee osteoarthritis: Comparisons with observation by spouses for construct validity. *Musculoskeletal Care*. 2017;15:263-271.
108. Peter WF, de Vet HCW, Terwee CB. Reliability of the Animated Activity Questionnaire for assessing activity limitations of patients with hip and knee osteoarthritis. *Musculoskeletal Care*. 2018;16:363-369.
109. Peter WF, Poolman RW, Scholtes VAB, de Vet HCW, Terwee CB. Responsiveness and interpretability of the Animated Activity Questionnaire for assessing activity limitations of patients with hip or knee osteoarthritis. *Musculoskeletal Care*. 2019;17:327-334.
110. Piva SR, Fitzgerald GK, Irrgang JJ, Bouzubar F, Starz TW. Get up and go test in patients with knee osteoarthritis. *Arch Phys Med Rehabil*. 2004;85:284-289.
111. Piva SR, Gil AB, Moore CG, Fitzgerald GK. Responsiveness of the activities of daily living scale of the knee outcome survey and numeric pain rating scale in patients with patellofemoral pain. *J Rehabil Med*. 2009;41:129-135.
112. Pollard B, Dixon D, Dieppe P, Johnston M. Measuring the ICF components of impairment, activity limitation and participation restriction: an item analysis using classical test theory and item response theory. *Health Qual Life Outcomes*. 2009;7:41.
113. Pollard B, Johnston M, Dieppe P. Exploring the relationships between International Classification of Functioning, Disability and Health (ICF) constructs of Impairment, Activity Limitation and Participation Restriction in people with osteoarthritis prior to joint replacement. *BMC Musculoskelet Disord*. 2011;12:97.

114. Ra HJ, Kim HS, Choi JY, Ha JK, Kim JY, Kim JG. Comparison of the ceiling effect in the Lysholm score and the IKDC subjective score for assessing functional outcome after ACL reconstruction. *Knee*. 2014;21:906-910.
115. Reid A, Birmingham TB, Stratford PW, Alcock GK, Giffin JR. Hop testing provides a reliable and valid outcome measure during rehabilitation after anterior cruciate ligament reconstruction. *Phys Ther*. 2007;87:337-349.
116. Rejeski WJ, Ettinger WH, Schumaker S, James P, Burns R, Elam JT. Assessing performance-related disability in patients with knee osteoarthritis. *Osteoarthritis and Cartilage*. 1995;3:157-167.
117. Roos EM, Roos HP, Lohmander LS, Ekdahl C, Beynnon BD. Knee Injury and Osteoarthritis Outcome Score (KOOS)--development of a self-administered outcome measure. *J Orthop Sports Phys Ther*. 1998;28:88-96.
118. Schmitt LC, Paterno MV, Huang S. Validity and internal consistency of the international knee documentation committee subjective knee evaluation form in children and adolescents. *Am J Sports Med*. 2010;38:2443-2447.
119. Sharma S, Wilson R, Pryymachenko Y, et al. Reliability, Validity, Responsiveness, and Minimum Important Change of the Stair Climb Test in Adults With Hip and Knee Osteoarthritis. *Arthritis Care Res (Hoboken)*. 2023;75:1147-1157.
120. Singh JA, Luo R, Landon GC, Suarez-Almazor M. Reliability and clinically important improvement thresholds for osteoarthritis pain and function scales: a multicenter study. *J Rheumatol*. 2014;41:509-515.
121. Steultjens MP, Dekker J, van Baar ME, Oostendorp RA, Bijlsma JW. Internal consistency and validity of an observational method for assessing disability in mobility in patients with osteoarthritis. *Arthritis Care Res*. 1999;12:19-25.
122. Steultjens MP, Roorda LD, Dekker J, Bijlsma JW. Responsiveness of observational and self-report methods for assessing disability in mobility in patients with osteoarthritis. *Arthritis Rheum*. 2001;45:56-61.
123. Stratford PW, Kennedy D, Pagura SM, Gollish JD. The relationship between self-report and performance-related measures: questioning the content validity of timed tests. *Arthritis Rheum*. 2003;49:535-540.
124. Stratford PW, Kennedy DM. A comparison study of KOOS-PS and KOOS function and sport scores. *Phys Ther*. 2014;94:1614-1621.
125. Stratford PW, Kennedy DM, Woodhouse LJ. Performance measures provide assessments of pain and function in people with advanced osteoarthritis of the hip or knee. *Phys Ther*. 2006;86:1489-1496.
126. Suwit A, Rungtiwa K, Nipaporn T. Reliability and Validity of the Osteoarthritis Research Society International Minimal Core Set of Recommended Performance-Based Tests of Physical Function in Knee Osteoarthritis in Community-Dwelling Adults. *Malays J Med Sci*. 2020;27:77-89.
127. Takacs J, Garland SJ, Carpenter MG, Hunt MA. Validity and reliability of the community balance and mobility scale in individuals with knee osteoarthritis. *Phys Ther*. 2014;94:866-874.
128. Takacs J, Krowchuk NM, Goldsmith CH, Hunt MA. Factor Analysis of the Community Balance and Mobility Scale in Individuals with Knee Osteoarthritis. *Physiother Res Int*. 2017;22:
129. Tanimura C, Morimoto M, Hiramatsu K, Hagino H. Difficulties in the daily life of patients with osteoarthritis of the knee: scale development and descriptive study. *J Clin Nurs*. 2011;20:743-753.
130. Tegner Y, Lysholm J. Rating systems in the evaluation of knee ligament injuries. *Clin Orthop Relat Res*. 1985;43-49.

131. Terwee CB, Coopmans C, Peter WF, et al. Development and validation of the computer-administered animated activity questionnaire to measure physical functioning of patients with hip or knee osteoarthritis. *Phys Ther*. 2014;94:251-261.
132. Tolk JJ, Janssen RPA, Prinsen CAC, et al. The OARSI core set of performance-based measures for knee osteoarthritis is reliable but not valid and responsive. *Knee Surg Sports Traumatol Arthrosc*. 2019;27:2898-2909.
133. Tubach F, Ravaud P, Baron G, et al. Evaluation of clinically relevant changes in patient reported outcomes in knee and hip osteoarthritis: the minimal clinically important improvement. *Ann Rheum Dis*. 2005;64:29-33.
134. van Meer BL, Meuffels DE, Vissers MM, et al. Knee injury and Osteoarthritis Outcome Score or International Knee Documentation Committee Subjective Knee Form: which questionnaire is most useful to monitor patients with an anterior cruciate ligament rupture in the short term? *Arthroscopy*. 2013;29:701-715.
135. Villadsen A, Roos EM, Overgaard S, Holsgaard-Larsen A. Agreement and reliability of functional performance and muscle power in patients with advanced osteoarthritis of the hip or knee. *Am J Phys Med Rehabil*. 2012;91:401-410.
136. Watson CJ, Propps M, Ratner J, Zeigler DL, Horton P, Smith SS. Reliability and responsiveness of the lower extremity functional scale and the anterior knee pain scale in patients with anterior knee pain. *J Orthop Sports Phys Ther*. 2005;35:136-146.
137. Wellsandt E, Axe MJ, Snyder-Mackler L. Poor Performance on Single-Legged Hop Tests Associated With Development of Posttraumatic Knee Osteoarthritis After Anterior Cruciate Ligament Injury. *Orthop J Sports Med*. 2018;6:2325967118810775.
138. Whitehouse SL, Crawford RW, Learmonth ID. Validation for the reduced Western Ontario and McMaster Universities Osteoarthritis Index function scale. *J Orthop Surg (Hong Kong)*. 2008;16:50-53.
139. Williams T, Burley D, Evans L, et al. The structural validity of the IKDC and its relationship with quality of life following ACL reconstruction. *Scand J Med Sci Sports*. 2020;30:1748-1757.
140. Williams VJ, Piva SR, Irrgang JJ, Crossley C, Fitzgerald GK. Comparison of reliability and responsiveness of patient-reported clinical outcome measures in knee osteoarthritis rehabilitation. *J Orthop Sports Phys Ther*. 2012;42:716-723.
141. Woon EL, Low J, Sng YL, Hor AB, Pua YH. Feasibility, correlates, and validity of the one-leg sit-to-stand test in individuals following anterior cruciate ligament reconstruction. *Phys Ther Sport*. 2021;52:280-286.
142. Yang KG, Raijmakers NJ, Verbout AJ, Dhert WJ, Saris DB. Validation of the short-form WOMAC function scale for the evaluation of osteoarthritis of the knee. *J Bone Joint Surg Br*. 2007;89:50-56.
143. Zamboti CL, Marçal Camillo CA, Ricardo Rodrigues da Cunha AP, Ferreira TM, Macedo CSG. Impaired performance of women with patellofemoral pain during functional tests. *Braz J Phys Ther*. 2021;25:156-161.
